# Development, validation and effectiveness of diagnostic prediction tools for colorectal cancer in primary care: a systematic review

**DOI:** 10.1186/s12885-020-07572-z

**Published:** 2020-11-10

**Authors:** Bogdan Grigore, Ruth Lewis, Jaime Peters, Sophie Robinson, Christopher J. Hyde

**Affiliations:** 1 Exeter Test Group, College of Medicine and Health, University of Exeter Medical School, Exeter, UK; 2 North Wales Centre for Primary Care Research, Bangor University, Bangor, UK; 3 Peninsula Technology Assessment Group, College of Medicine and Health, University of Exeter Medical School, Exeter, UK

**Keywords:** Cancer, Primary care, Diagnostic prediction models

## Abstract

**Background:**

Tools based on diagnostic prediction models are available to help general practitioners (GP) diagnose colorectal cancer. It is unclear how well they perform and whether they lead to increased or quicker diagnoses and ultimately impact on patient quality of life and/or survival. The aim of this systematic review is to evaluate the development, validation, effectiveness, and cost-effectiveness, of cancer diagnostic tools for colorectal cancer in primary care.

**Methods:**

Electronic databases including Medline and Web of Science were searched in May 2017 (updated October 2019). Two reviewers independently screened titles, abstracts and full-texts. Studies were included if they reported the development, validation or accuracy of a prediction model, or assessed the effectiveness or cost-effectiveness of diagnostic tools based on prediction models to aid GP decision-making for symptomatic patients presenting with features potentially indicative of colorectal cancer. Data extraction and risk of bias were completed by one reviewer and checked by a second. A narrative synthesis was conducted.

**Results:**

Eleven thousand one hundred thirteen records were screened and 23 studies met the inclusion criteria. Twenty-studies reported on the development, validation and/or accuracy of 13 prediction models: eight for colorectal cancer, five for cancer areas/types that include colorectal cancer. The Qcancer models were generally the best performing.

Three impact studies met the inclusion criteria. Two (an RCT and a pre-post study) assessed tools based on the RAT prediction model. The third study looked at the impact of GP practices having access to RAT or Qcancer.

Although the pre-post study reported a positive impact of the tools on outcomes, the results of the RCT and cross-sectional survey found no evidence that use of, or access to, the tools was associated with better outcomes. No study evaluated cost effectiveness.

**Conclusions:**

Many prediction models have been developed but none have been fully validated. Evidence demonstrating improved patient outcome of introducing the tools is the main deficiency and is essential given the imperfect classification achieved by all tools. This need is emphasised by the equivocal results of the small number of impact studies done so far.

**Supplementary Information:**

The online version contains supplementary material available at 10.1186/s12885-020-07572-z.

## Background

Colorectal cancer is the third most frequent cancer and the second leading cause of cancer-related death in the world [1]. In 2014–2016 there were 42,042 new cases of colorectal cancer in the UK, with 57% of people with colorectal cancer surviving for 10 years or more [2].

Research suggests that cancer prognosis can be improved by reducing the time to diagnosis [3], as earlier diagnosis is associated with earlier stage at diagnosis [4], and earlier treatment is associated with improved survival [5]. Reducing time to diagnosis also has the potential to reduce presentation via emergency admissions, and prevent the poorer survival associated with that route of diagnosis [6]. A national cancer screening programme exists in the National Health Service (NHS) for colorectal cancer, and the National Awareness and Early Diagnosis Initiative (NAEDI) (to increase public awareness on the signs and symptoms of cancer [7]) is intended to improve early diagnosis. However, as many individuals go through primary care as a route for diagnosis [6], so efforts here could improve cancer survival.

Cancer diagnosis in primary care is not straightforward. Symptoms of cancer are commonly seen but mostly have non-cancer origins [8]. Of those individuals referred from primary care via the two-week wait (2WW) referrals for suspected colorectal cancer in areas of England, approximately 5–8% were ultimately diagnosed with cancer [9, 10]. The type and presence of symptoms can vary greatly [11] and it is not surprising that patients can have multiple general practitioner (GP) consultations before being referred, especially for those cancers that have less well-known signs and symptoms [12]. Thus, tools to help improve cancer diagnosis in primary care have great potential to impact on diagnoses and subsequent treatment options, leading to better outcomes for patients.

Diagnostic prediction models combine multiple predictors, such as symptoms and patient characteristics, to obtain the risk of the presence or absence of a disease within an individual patient [13, 14]. These prediction models can then be used to develop diagnostic tools (such as a website risk calculator, or mouse mat containing estimates of risk depending on features) to assist doctors in estimating probabilities and potentially influence their decision making [14]. To evaluate diagnostic prediction models, there are three important stages, or types of studies: prediction model development, prediction model validation, and assessment of the impact of prediction models in practice (generally implemented as diagnostic tools). The first two are often conducted as part of the same study, and are generally evaluated using a single cohort design. These types of studies are commonly found in the diagnostic prediction literature, with some studies also reporting results of an external validation [15]. To assess the impact of the prediction model (the third stage), comparative studies are required to evaluate the ability of the tool to guide patient management. However, very few diagnostic prediction models that are developed go on to be evaluated for their clinical impact [15] or cost-effectiveness.

Tools currently available to GPs in the UK to help cancer diagnosis, beyond the National Institute for Health and Care Excellence (NICE) guidelines for suspected cancer referral [8], are based on diagnostic prediction models, and are integrated into GP software systems.

1. The Risk Assessment Tool (RAT) developed by Hamilton and colleagues which provides estimates of cancer risk for 17 cancers based on symptoms alone is integrated into Vision (INPS), and
2. The Qcancer tool, which estimates the risk of 11 cancers based on symptoms and patient characteristics, and overall cancer risk in males and females, is integrated into EMIS Web.

There is recent evidence that these tools are being used in primary care [16], however it is unclear whether these tools impact on GP decision-making, and ultimately on patient outcomes.

Systematic reviews have looked at the use of prediction models for colorectal cancer in primary and secondary care [17]. However, more research in the primary care setting had been published for colorectal cancer since, so we sought to systematically review this evidence. The aim of our review was to identify reports on the development, validation or accuracy of prediction models, as well as evidence evaluating the impact (i.e. effectiveness or cost-effectiveness) of symptom-based diagnostic tools that could be used to inform colorectal cancer diagnosis decision-making in primary care.

## Methods

This systematic review was conducted as part of a wider programme considering risk assessment tools for any cancer site [18]. Protocols relevant to the systematic review described here were registered on PROSPERO (CRD42017068373, CRD42017068375).

The systematic review was conducted in accordance with good practice guidelines [19] and is reported here in line with the Preferred Reporting Items for Systematic Reviews and Meta-Analyses (PRISMA) guidelines [20].

### Search strategy

Bibliographic searches of relevant databases (Medline, Medline in Process, Embase, Cochrane, Web of Science), were conducted in May 2017 and updated in October 2019.

The search strategies were developed by an information specialist (SR) and comprised terms for cancer, terms for primary care, terms for decision support tools and terms for diagnosis (see.supplementary Table S1). No date, language or other limits were used. Search filters for clinical prediction models were investigated but none were thought to be fully tested or reliable. A balance was sought between sensitivity of search results and volume of papers to screen. As the search strategies were originally developed to identify reports related to prediction models for any cancer site [18], no cancer site specific terms were used. Instead, we retrospectively excluded non-colorectal cancer studies from the current systematic review.

The search results were exported to Endnote X7 (Thomson Reuters, NY, USA) and de-duplicated using automatic and manual checking.

Additional searches were conducted using Scopus (Elsevier) on the references, as well as any citations of the items included after full-text screening, in order to identify additional relevant studies. Searches were also conducted for identified named tools (QCancer, RAT, CAPER, Bristol-Birmingham equation) in order to ensure search results were sufficiently comprehensive.

### Inclusion and exclusion criteria

Diagnostic prediction models are defined as multivariate statistical models that predict the probability or risk that a patient currently has cancer based on a combination of known features of that patient, such as symptoms, signs, test results and patient characteristics [21]. Symptoms could be self-reported by the patient, or prompted by physician’s questioning. Signs and test results are identified within primary care via routine testing (such as full blood count, urine dipstick testing, clinical signs), as are patient characteristics (socio-demographic variables, personal and family history). Studies that simply looked at ‘red-flag symptoms’ or symptom lists and (weighted) scores that did not provide a numerical risk of current cancer were excluded. Models developed with secondary care data (i.e. referred patients) were only included if an attempt was made to validate the models with primary care data.

Inclusion and exclusion criteria are presented in Table 1.

**Table 1:** Inclusion and exclusion criteria

**Population**	Included: adult symptomatic patients (with symptoms being indicative of cancer) presenting at primary care or patients referred with symptoms indicative of cancer
	Excluded: asymptomatic patients (screening population).
**Technology**	Included: Diagnostic prediction models, based on 2 or more features^a^, that estimate the risk of prevalent but undiagnosed colorectal cancer.
	Excluded: prognostic or screening prediction models Statistical tools that estimate the probability of developing cancer over a defined period of time. Prediction models that did not include colorectal cancer.
**Setting**	Included: primary care
	Excluded: secondary care; on-line tools developed for use by the general population
**Study design**	Included: - any design for the ***development, validation or accuracy*** of diagnostic prediction models (as defined under ‘Technology’); - comparative studies of diagnostic tools that assessed ***impact*** in clinical practice (Randomised controlled trials, controlled before-after, and interrupted time-series; studies analysing national trends in cancer diagnosis before and after diagnostic tools became available)
	Excluded: uncontrolled studies reporting qualitative data
**Comparison**	Usual care or the use of another diagnostic tool
**Outcomes**	**For studies reporting development, validation and/or accuracy of prediction models:** Estimates of the risk of being diagnosed with cancer (e.g. ORs, HRs) AND/OR Any details on the development, validation or accuracy of the tool: • *Model development:* method; assumptions; predictors; shrinkage; coefficient weighting • *Model evaluation (validation)* • *Assessing (quantifying) model performance:* discrimination (ability to discriminate participants with or without the outcome, e.g. area under the ROC curve); calibration (agreement between predicted and observed outcome); overall performance (for discrimination and calibration, e.g. R2); classification (e.g. sensitivity, specificity, predictive values) **For studies reporting evaluations of the impact of tools:** *Primary outcomes* - patient-related outcome measures (including the number of cancer diagnoses, time to cancer diagnosis, stage of cancer at diagnosis, resection rates, patient health-related quality of life, other patient-reported outcome measures); - survival; - economic outcome measures (resource use, cost per diagnosis), cost per QALY; *Secondary outcomes* - referral patterns.
	Exclude: models that report the risk of survival (or stage at diagnosis etc.)
**Publication type**	Included: Published in full and in English
	Excluded: commentaries, letters

*Abbreviations*: *HR* Hazard ratio, *N/A* Not applicable, *OR* Odds ratio, *QALY* Quality-adjusted life year, *ROC* Receiver operating characteristic

Note: ^a^ Features include symptoms and other information, such as elicited signs, patient characteristics and test results

### Selection of studies

Titles and abstracts were screened for relevance independently (by BG and RL), and any disagreements were resolved by consensus. Pilot screening was undertaken for the first 100 hits to ensure both reviewers were interpreting the inclusion and exclusion criteria in the same way. Articles retained were obtained in full and further screened independently by the two reviewers. For any disagreements that were not resolved, a third reviewer (CH) made the final decision.

The development and validation aspects of particular prediction models were often reported in multiple studies (e.g. the development and internal validation of the Q cancer prediction model was presented in one paper by Hippisley-Cox and colleagues, 2012 [22] and the external validation in a separate paper (Collins and colleagues, 2012 [23]) All studies related to the same specific prediction model were collated regardless of whether they refer to the development, validation and/or impact of that tool.

### Data extraction

To extract relevant data from each included study, standardised data extraction forms were used that evolved following piloting and discussion among reviewers. One reviewer (BG) extracted the data, which was checked by a second reviewer (RL). The following data were extracted from all study types: included cancer type(s), study design, country, sample size, participant recruitment (with inclusion and exclusion criteria) and participant characteristics. For studies reporting on the development and/or validation of prediction models an adaptation of the CHARMS checklist (CHecklist for critical Appraisal and data extraction for systematic Reviews of prediction Modelling Studies) [24] was used to extract additional relevant data, including data source, number of participants with specific cancer, features of the model (what symptoms, test results, patient demographics etc. are included), how features are defined and measured, definition of primary and secondary outcomes, how and when outcomes are assessed, main results (including model performance, validation and estimates of risk), features included in final model. For studies reporting the impact of tools based on prediction models additional items extracted included characteristics of the tool (including whether based on symptoms alone or other features in addition to symptoms), definition of outcomes, main results including confidence intervals, and subgroup analyses, where available.

### Risk of bias assessment

Risk of bias of studies reporting the development and/or validation of prediction models was assessed with the PROBAST [25] (Prediction model Risk of Bias ASsessment Tool) checklist. The derived checklist assesses the risk of bias and applicability of prediction-modelling studies on 5 domains: participant selection, predictors, outcome, sample size and missing data, analysis.

For studies reporting on the impact of tools based on decision models a risk-of-bias form based on the Cochrane EPOC (Effective Practice and Organisation of Care) group recommendations [26] was used. All risk of bias assessments were conducted by one reviewer (BG) and checked by a second reviewer (RL).

### Synthesis

Owing to the heterogeneity between included studies a narrative review of the studies was conducted.

## Results

### Studies identified

Search phrases were finalised and searches were run in May 2017. A total of 9352 records were obtained through database searching. Additional reference and citation searches on tool names resulted in another 4171 records. After de-duplication, 9780 records were obtained. The database searches were updated in October 2019, and resulted in 2254 additional new records (after de-duplication). After screening the title and abstracts of these records independently by two reviewers, 260 records were retained for full text screening.

**Fig. 1:**
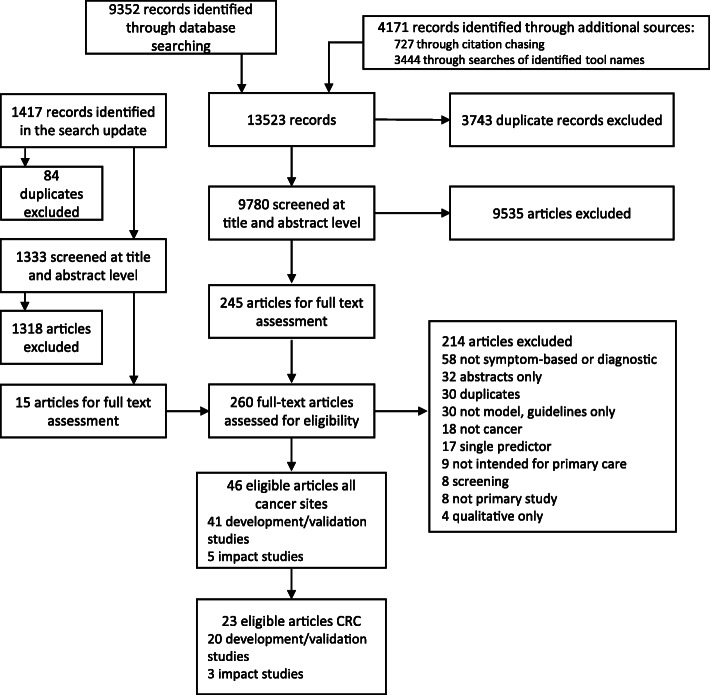
PRISMA diagram of the included studies. Abbreviations: CRC = colorectal cancer

We identified two systematic reviews. Scanning their reference list led to the inclusion of two additional studies not found in the database search. One systematic review also included validation of models [27]. In the end, 23 records were identified that were relevant for colorectal cancer (Fig. 1).

Discussions with collaborators led to the identification of relevant grey literature, but no such studies were deemed eligible for inclusion.

#### Development/validation studies

Elias and colleagues (2017) [27] aimed to identify and validate published diagnostic models to safely reduce unnecessary endoscopy referrals for colorectal cancer. A systematic review of the literature was undertaken up until 2015 and identified models were validated using a cross-sectional Dutch dataset referred to as CEDAR (*n* = 810). The definition of model used by Elias and colleagues is very broad and includes guidelines and weighted scores. Therefore, although Elias and colleagues identified 18 models, only four are relevant to our review: Fijten and colleagues (1995) [28] and Marshall and colleagues (2011) [29] which were identified from our searches, while Muris and colleagues (1995) [30] and Nørrelund and colleagues (1996) [31] are new inclusions. Due to the fact that Elias and colleagues attempted to validate the models they found, their validation of these four models is included in the results below.

Of the 20 included model development or validation studies, 17 report on the development (with some also reporting on validation) of models, four only report model validation.

#### Prediction models

The included studies (excluding the validation by Elias and colleagues [27]) reported on 13 different prediction models. Eight models are specifically for colorectal cancer: the Bristol-Birmingham equation (Marshall [29]), a Dutch model (Fijten [28]), a machine learning algorithm (Kop [32]), a Danish model (Nørrelund [31]), Qcancer (Hippisley-Cox [22]), RAT 2005 (Hamilton [33]), RAT 2009 (Hamilton [34]) and RAT 2017 (Stapley [35]). One model relates to metastatic cancer (RAT, Hamilton [36]), and the remaining four models cover multiple cancer sites which include colorectal cancer: Qcancer for males (Hippisley-Cox [37]), Qcancer for females (Hippisley-Cox [38]), a model for abdominal complaints (Muris [30]), and a model for abdominal cancers (Holtedahl 2018 [39]). Elias [27] and Collins [23] reported on the validation of one or more of the above models.

Table 2 provides a brief description of the models, their stages of development, the cancer sites covered (colorectal cancer-specific or other) and study designs.

**Table 2:** Summary of the prediction models, their stages of development, the cancer sites covered and study designs

Prediction model	Number and category of descriptors	Stage of development	Study design	Country	Population	Source
**Colorectal cancer**						
Bristol-Birmingham equation	8 Symptoms, Test results	External validation	Retrospective Case-control	UK	Derivation cohort: THIN Validation cohort: CAPER	Marshall 2011 [29]
		External validation	Prospective cohort	The Netherlands	CEDAR study: Patients referred to endoscopy centres by participating Dutch primary care practices. 2009–2012	Elias 2017 [27]
Netherlands model	3 Symptoms, Patient demographics	Apparent performance	Prospective cohort	The Netherlands	290 consecutive patients with rectal bleeding presenting to 83 GPs in Limburg (Netherlands) September 1988 to April 1990Predictors: Questionnaires completed by GPs and patients, and laboratory test results.	Fijten 1995 [28]
		External validation	Prospective cohort	UK	patients referred from primary care with colorectal symptoms over a 3-yr period to the Leighton Hospital, Crewe, Cheshire, UK	Hodder 2005 [40]
		External validation	Prospective cohort	Netherlands	CEDAR study: Patients referred to endoscopy centres by participating Dutch primary care practices. 2009–2012	Elias 2017 [27]
Machine learning algorithm	Numerous models are reported Patient demographics, Symptoms, Medical history, Test results	Apparent performance	Case-control	The Netherlands	anonymised electronic records from two GP database systems from the Utrecht region, Netherlands, between 01 and 07-2006 and 31-12-2011	Kop 2015 [41]; Kop 2016 [32]; Hoogendoorn 2015 [42]
Danish model	2 Patient demographics Symptoms	Apparent performance	Prospective cohort	Denmark	Patients presenting to GPs with first episode of rectal bleeding. Study 1: 750 GPs 1989–1991 Study 2: 450 GPs 1991–1992	Nørrelund 1996 [31]
		External validation	Prospective cohort	The Netherlands	CEDAR study: Patients referred to endoscopy centres by participating Dutch primary care practices. 2009–2012	Elias 2017 [27]
Qcancer	6 (females) 7 (males) Symptoms, Medical history, Test results	Internal validation	open Prospective cohort	UK	QResearch database	Hippisley-Cox 2012c [22]
		External validation	Prospective cohort	UK	THIN database	Collins 2012 [23]
RAT (2005)	10 Symptoms, Test results	Apparent performance	Case-control	UK	Patients attending all 21 general practices in Exeter, Devon, UKCases identified from the cancer registry at the Royal Devon and Exeter Hospital	Hamilton 2005 [33]
		External validation	Prospective cohort	The Netherlands	CEDAR study: Patients referred to endoscopy centres by participating Dutch primary care practices. 2009–2012	Elias 2017 [27]
RAT (2009)	8 Symptoms, Test results	Apparent performance	Case-control	UK	THIN database	Hamilton 2009 [43]
RAT (bowel)	10 Symptoms, Test results	Apparent performance	Case-control	UK	GPRD (currently called the CPRD)	Stapley 2017 [35]
**Metastatic cancer**						
RAT	7 Symptoms, Test results	Apparent performance	Case-control	UK	Patients attending 11 general practices in Devon, UK	Hamilton 2015 [36]
**Multiple cancer sites**						
Qcancer (female)	7 (uterine) 10 (breast, blood) 11 (ovarian, renal) 12 (cervical) 13 (colorectal, gastro-oesophageal) 14 (pancreatic) 15 (lung) 22 (other cancers) Medical history, Symptoms, Test results, Patient demographics	Internal validation	Open prospective cohort	UK	QResearch database	Hippisley-Cox 2013 [38]
QCancer (male)	3 (testicular) 8 (renal tract) 12 (colorectal) 13 (gastro-oesophageal) 14 (prostate, blood) 15 (pancreatic) 17 (lung) 20 (other cancers) Medical history, Symptoms, Test results, Patient demographics	Internal validation	Open prospective cohort	UK	QResearch database	Hippisley-Cox 2013b [37]
Muris abdominal complaints model	5 Symptoms Patient demographics Test results	Apparent performance	Prospective cohort	The Netherlands	Patients presenting to GPs for new abdominal complaints. 1989	Muris 1995 [30]
(Netherlands)		External validation	Prospective cohort	The Netherlands	CEDAR study: Patients referred to endoscopy centres by participating Dutch primary care practices. 2009–2012	Elias 2017 [27]
Abdominal model, Holtedahl and colleagues (2018)	4 Symptoms, Patient demographics	Apparent performance	Prospective cohort	Norway, Denmark, Sweden, Scotland, Belgium, Netherlands	GP records from the participating countries	Holtedahl, 2018 [39]

*Abbreviation*: *RAT(s)* Risk assessment tool(s)

The risk prediction models referred to as RATs [33, 35, 36, 43, 44] were designed to be used with patients presenting to primary care with “low-risk-but-not-no-risk symptoms” [45]. Early versions of RATs were developed using case–control data from Devon, UK as part of the CAPER studies [34]. Later models were derived using UK-wide primary care data – the Clinical Practice Research Datalink (formerly General Practice Research Database) [35, 44, 46–51], and The Health Improvement Network (THIN) database [43, 52]. In addition to the models identified in this systematic review as relevant to colorectal cancer, RATs exist for the following cancer sites: lung, ovarian, kidney, bladder, pancreas, breast, uterine, brain, prostate, Hodgkin lymphoma, non-Hodgkin lymphoma and multiple myeloma. The RATs are available as prints on common office objects (e.g. mousepads) and are integrated into general practitioner software in the form of the electronic Cancer Decision Support (eCDS). Regardless of the format, they provide risk estimates for patients with single symptoms of possible cancer, pairs of symptoms and repeat attendances with the same symptoms. Elias used a Dutch dataset to externally validate the 2005 colorectal version of RATs [27]. No other RAT was externally validated.

The QCancer series of models can be used both in symptomatic (diagnostic models) and asymptomatic (prognostic models) patients [53]. QCancer was developed in the QRESEARCH database, a large database comprising over 12 million anonymised health records from 602 general practices throughout the United Kingdom using the EMIS (Egton Medical Information Systems) computer system. Initially, several models were developed for each cancer type in symptomatic populations, in addition to colorectal: lung, renal, gastro-oesophageal, pancreatic and ovarian cancer. An updated approach incorporates multiple risk factors and symptoms into one model for males and one model for females to predict cancer risk. Most of these models have been externally validated in UK-wide populations (e.g. THIN database [54]). QCancer is available as an online calculator (www.qcancer.org), which provides estimates of absolute risk of any cancer with a breakdown of type of cancer based on both risk factors such as age, gender and family history, which increase the likelihood of cancer, and risk markers such as haemoptysis or features, usually symptoms (e.g. weight loss), suggesting that cancer is already present.

Marshall and colleagues (2011) used data from the THIN dataset (> 40,000 participants) to construct a model for colorectal cancer, known as the Bristol-Birmingham equation [29]. The model was validated by Marshall et al. using the UK CAPER dataset and was also validated by Elias et al. (26) in a Dutch population. Data from 290 patients presenting to GPs in the Netherlands with rectal bleeding (from 1988 to 1990) were used by Fijten and colleagues (1995) [28] to develop a prediction model for colorectal cancer (Netherlands model). The Netherlands model was validated by Hodder and colleagues (2005) [40] using secondary care data from the UK, and by Elias and colleagues (2017) [27] using a Dutch dataset. Kop and colleagues (2015) [32, 41, 42] used a machine learning algorithm to develop a prediction model for colorectal cancer using electronic records of almost 220,000 patients from two GP practices in the Netherlands. We found no external validation of this model. A Danish colorectal model [31] has also been developed for use in primary care, this was externally validated using a Dutch dataset by Elias and colleagues (2017) [27].

Muris and colleagues (1995) [30] developed a model using data from the Netherlands to predict multiple cancers related to abdominal complaints, which was externally validated by Elias [27].

Holtedahl and colleagues (2018) [39] detail the development of a prediction model for abdominal cancers. These are defined as all cancers of the digestive organs, female genital organs and urinary organs (including testis). Data on 61,802 patients, recorded during GP consultations over a 10 day period from Norway, Denmark, Sweden, Scotland, Belgium, and the Netherlands, were used to develop the model. No validation of the model was identified.

The models are in various stages of development. A total of 5 models (or versions of models) have only assessed apparent performance [35, 36, 39, 41, 43], two models have been internally validated (Qcancer for males and Qcancer for females), one model was updated as a result of using a different data source [43]. One of the four Qcancer versions [22], one RAT version [33] and four of the other prediction models [28–31] have been externally validated, the highest level of evidence identified in this systematic review. Apart from the two Qcancer versions, which were externally validated by Collins and Altman, all other external validations were conducted by Elias et al. [27]. This was a systematic review which used a cross-sectional Dutch dataset referred to as CEDAR (*n* = 810) to validate the models they identified.

All of the models were developed in primary care settings in Europe. Only five models were not derived from UK-only data: Fijten and colleagues (1995) [28], Kop and colleagues (2015) [41], and Muris and colleagues (1995) [30] were developed in the Netherlands, Nørrelund and colleagues (1996) [31] was developed in Denmark, and Holtedahl which used data from Norway, Denmark, Sweden, Scotland, Belgium and the Netherlands. For those models having been externally validated, most were validated in the country in which it was developed except for: the validation [40] of the Netherlands colorectal cancer model [28] in a UK population, the validation of the Danish colorectal cancer [31] in a Dutch population [27] and the validation of the colorectal version of RATs (UK) [33] in a Dutch population [27].

#### Critical appraisal

The assessment of risk of bias is summarised in Table 3, and given in more detail in supplementary Table S3. Note that for the RATs and Qcancer models, only one entry each is shown as all versions of the RAT or Qcancer model scored the same for each aspect of the risk of bias tool used. Qcancer development and validation studies were judged to be of low risk of bias. For the RAT development studies, there is uncertainty as to the risk of bias for how predictors and sample size and participants were dealt with, and a high risk of bias concerning the analysis. For the development of the other models, risk of bias was variable across all domains, although most models have a low risk of bias with respect to how outcomes are dealt with.

**Table 3:** Risk of bias assessment for the included model development/validation studies

Model (author offirst version)	Stage of development covered	I. Participantselection^a^	II. Predictors^a^	III. Outcome^a^	IV. Samplesize andparticipantflow^a^	V. Analysis^a^
RAT (Hamilton) series of models for colorectal and meta-staticcancer [33, 36, 43]}						
	Apparent performance	✓	?	✓	?	x
	External validation (colorectal only) [27]	✓	✓	✓	?	?
QCancer (Hippisley-Cox) series of models for **colorectal andmultiple sites for females and males** [22, 23, 37, 38]						
	Internal validation	✓	✓	✓	✓	✓
	External validation (colorectal only) [23]	✓	✓	✓	✓	✓
Bristol-Birmingham (Marshall) [29] model for **colorectal cancer**						
	External validation	✓	?	✓	?	✓
	External validation (Elias and colleagues,2017) [27]	✓	✓	✓	?	?
Netherlands’ (Fitjen 1995 [28]) model for **colorectal cancer**						
	Apparent performance	x	✓	✓	?	x
	External validation (Hodder and colleagues,2005) [40]	x	?	x	✓	?
	External validation (Elias and colleagues,2017) [27]	✓	✓	✓	?	?
Netherlands’ (Kop) [32] ‘machine learning’ for **colorectal cancer**						
	Apparent performance	✓	?	✓	?	?
Danish (Nørrelund 1996 [31]) model for **colorectal cancer**						
	Apparent performance	✓	?	✓	?	x
	External validation (Elias and colleagues,2017) [27]	✓	✓	✓	?	?
Netherlands’ (Muris 1995 [30]) model for **abdominal complaints**						
	Apparent performance	?	✓	✓	?	x
	External validation (Elias and colleagues,2017) [27]	✓	✓	✓	?	?
Prediction model for **abdominal cancers**(Holtedahl and colleagues, 2018) [39]						
Holtedahl, 2018	Apparent performance	?	✓	?	x	?

*Abbreviations*: *RAT (s)* Risk assessment tool(s), *SR2* Systematic review 2

Notes:^a^multiple ordered by stage of development if different

Key: ✓, low risk of bias; x, high risk of bias; ?, unclear risk of bias

The external validation of the colorectal cancer RAT, and of many of the other models by Elias was judged to be of uncertain risk of bias for how sample size and patient flow was dealt with, and how analyses were conducted.

Overall, apart from the Qcancer studies, the risk of bias of the development and validation studies is mixed and/or uncertain.

#### Performance of the models

As with many systematic reviews of prediction models, we found a mix of outcomes reported on the different models. The most widely reported outcome was the area under the curve (AUC). AUC estimates were calculated from external datasets for seven of the 13 models (Table 4). As some authors reported AUCs based on the model derivation dataset, in Table 4 we distinguishing between whether the reported AUC is estimated using the derivation dataset, or the external dataset. Note that for the remaining six models, which includes three of the RATs, we could find no external validation of any kind.

**Table 4:** Available AUC estimates (and 95% confidence intervals) for the prediction models

Prediction model	Validation (using derivation or external dataset)	Dataset used, country	AUC (95% CI)	Source
Colorectal cancer				
Bristol-Birmingham equation [29]	Derivation	THIN, UK	0.83 (0.82, 0.84)	[29]
	External	CAPER, UK	0.92 (0.91, 0.94)	[29]
	External	CEDAR, Netherlands	0.84 (0.77, 0.90)	[27]
Netherlands model [28]	Derivation	Primary care, Netherlands	0.97	[28]
	External	Secondary care, UK	0.78 (0.74, 0.81)	[40]
	External	CEDAR, Netherlands	0.72 (0.62, 0.81)	[27]
Netherlands model including polyps [28]	Derivation	Primary care, Netherlands	0.92	[28]
Qcancer (male) [22]	Derivation	Qresearch, UK	0.91 (0. 09, 0.91)	[22]
	External	THIN (multiple imputation), UK	0.92 (0.91, 0.92)	[23]
		THIN (complete case analysis), UK	0.90 (0.89, 0.91)	[23]
Qcancer (female) [22]	Derivation	Qresearch, UK	0.89 (0.88, 0.90)	[22]
	External	THIN (complete case analysis), UK	0.91 (0.90, 0.92)	[23]
Danish model [31]	External	CEDAR, Netherlands	0.6 (0.48, 0.72)	[27]
RAT (2005) [33]	External	CEDAR, Netherlands	0.81 (0.75, 0.88)	[27]
Multiple cancer sites				
Muris abdominal complaints model [30]	External	CEDAR, Netherlands	0.62 (0.54, 0.70)	[27]

The Qcancer models are associated with the highest estimated AUC value from external validation: 0.92 (0.91, 0.92) and 0.91 (0.90, 0.92) for the male and female versions of the colorectal Qcancer model.

The Bristol-Birmingham equation was also associated with a high AUC value for external validity, but only in one of two studies. The two AUCs from external validation of the Bristol-Birmingham equation differ, with the AUC estimate from the UK CAPER dataset being much higher (0.92 (0.91, 0.94)) than that from the external validation using the Dutch CEDAR dataset (0.84 (0.77, 0.90)) or the derivation dataset (0.83 (0.82, 0.84)) [27, 29].

The Netherlands model for colorectal cancer was associated with the highest AUC score for internal validation (0.97), but this was not replicated when the model was used in a different population. The AUC value was much lower in both external validation studies, using either secondary care data from the UK (0.78 (0.74, 0.81)) or Dutch dataset (0.72 (0.62, 0.81)).

The remaining models are estimated to have mean AUCs between 0.6 and 0.8, with the Danish model for colorectal cancer and the Muris abdominal complaints model being the two lowest performing models. The only RAT for which an AUC is reported is for the 2005 version of the colorectal model from Elias [27], and is much lower than those from the Qcancer models, 0.81 (0.75, 0.88).

Estimates of NPV, PPV, sensitivity and specificity are available from the external validations by Elias of the Bristol-Birmingham equation [29], the models by Fijten [28], Nørrelund [31], and Muris [30] and the 2005 colorectal RAT [33] . Collins and Altman [23] also report these estimates for validation of the colorectal Qcancer model (see supplementary Table S2). The (male and female) colorectal Qcancer models are the only models to have estimates of sensitivity > 0.9 and specificity > 0.7. The 2005 colorectal RAT has a reported sensitivity of 0.95 and specificity of 0.45. The other four models (Bristol-Birmingham, Fijten, Nørrelund and Muris) all have high sensitivity (> 0.95), but very low specificity: 0.06 for Nørrelund to 0.36 for the Bristol-Birmingham equation. Marshall [29], Holtedahl [39], Hamilton [33], Hamilton [43] and Stapley [35] also report likelihood ratios (LRs), see Supplementary Table S2c. Marshall [29] report a LR of 14.7 for the Bristol-Birmingham equation, while the other 4 studies only report LRs for individual symptoms included in the model. These range from < 2 for some symptoms in the model reported in Hamilton [43] to > 30 for rectal bleeding in the model reported by Stapley [35].

#### Impact studies

Three studies were identified that attempted to evaluate the impact of tools based on diagnostic prediction models used in practice: a cross-sectional survey [16], a pre-post study [55] and a randomised controlled trial [56]. The RCT and pre-post studies evaluated the use of a combination of tools which included RATs for colorectal cancer. The cross-sectional survey by Price [16] evaluated the impact of GP practice access to RAT and/or Qcancer, see Table 5.

**Table 5:** Description of tools assessed in the three impact studies

Study ID	Prediction tool	Country of tool development	Tool description
Hamilton and colleagues*,* 2013 [55]	RAT presented on a mouse mat and desk top flip chart (for lung and colorectal cancer)	UK	The RAT algorithm is displayed in a table/matrix format, which allows a risk estimate to be calculated for a single symptom, pairs of symptoms or repeat attendances with the same symptom. The values are colour-coded to aid interpretation.
Emery and colleagues, 2017 [56]	Education resource card containing the RAT and referral guidelines	UK (RAT), Australia (guidelines)	Resource card containing the RAT tables for colorectal, lung and prostate cancer, as well as the Australian National Breast and Ovarian Cancer Centre guidelines for investigating new breast symptoms
Price and colleagues 2019 [16]	RAT and/or QCancer in any form (e.g. paper, software etc.) for any cancer	UK	Any affirmative GP practice access to RAT and/or QCancer

*Abbreviations*: *ID* Identification, *RAT(s)* Risk assessment tool(s)

Price and colleagues [16] compared UK practice-level 2WW referral rates between GP practices that reported access to RAT and/or Qcancer, with practices that reported no access to these two tools. The tools included Qcancer and RAT for any cancer, and the analyses were not restricted to colorectal cancer.

Hamilton and colleagues (2013) [55] investigated the number of times two RATs [34] – one for lung and one for colorectal cancer – were used, together with the number of subsequent referrals and investigations, before and 6 months after the introduction of the tools in general practice in the UK.

Emery and colleagues (2017) [56] evaluated the impact of two complex interventions in rural Australia – a GP intervention and a cancer awareness campaign – in a 2 × 2 design trial, compared to control groups. The GP intervention consisted of an “education resource card” that included RATs for colorectal, lung and prostate cancer, together with summaries of relevant guidelines for colorectal, lung and prostate cancer, with the addition of guidelines for breast cancer and training on the use of these resources. The RATs were based on diagnostic prediction models developed using a patient cohort from the UK [34]. Emery and colleagues (2017) [56] used the total diagnostic interval (TDI), i.e. the time from first symptom to cancer diagnosis, as an outcome measure.

#### Critical appraisal

The RCT by Emery was found to be at low risk of bias (see Table 6). Given the observational nature of the studies by Hamilton and Price [16], there are a number of concerns regarding their risk of bias.

**Table 6:** Risk of bias assessment for the three impact studies

	Randomsequencegeneration	Allocationconcealment	Baselineoutcomemeasurementssimilar	Baselinecharacteristicssimilar	Incompleteoutcomedata	Knowledge ofthe allocatedinterventionsadequatelypreventedduring the study	Protectionagainstcontamination	Selectiveoutcomereporting	Other risksof bias
**Randomised controlled trials**									
Emery 2017 [56]	✓	x	✓	✓	✓	✓	✓	✓	✓
**Pre-post study**									
Hamilton2013 [55]	N/A	N/A	N/A	N/A	?	N/A	N/A	?	x
**Cross-sectional survey**									
Price andcolleagues2019 [16]	N/A	N/A	N/A	?	?	✓	N/A	✓	x

Abbreviations: *N/A* Not applicable. Key: ✓, low risk; x, high risk; ?, unclear risk

#### Study outcomes

Emery and colleagues [56] did not find significant differences in the median or log-transformed (ln) mean time to diagnosis at either intervention level (community intervention vs control, GP intervention vs control) or when analysed by factorial design, tumour group or sub-intervals of the TDI.

Hamilton and colleagues (2013) [55] reported on changes in investigations carried out and rapid referrals before and after the introduction of the tools. They found a 26% increase in referrals for colorectal cancer and a 15% increase in GP requests for colonoscopies after introduction of the tools. However, only absolute numbers are reported, without data on total numbers of patients and GP visits, or the appropriateness of the referral.

Price and colleagues [16] did not find any differences in mean 2WW referral rates between practices reporting access to cancer decision-making tools and those who did not: mean difference in referral rate of 3.1 per 100,000 population (95% CI of − 5.5, 11.7). As the study considered RATs and Qcancer for any suspected cancer and 2WW referral rates for any cancer, the specific impact of colorectal cancer-relevant RATs or Qcancer tools on referrals for colorectal cancer cannot be evaluated.

Study results are summarised in Table 7.

**Table 7:** Results reported by the impact studies

Study ID	Prediction tool	Country	Study design	Intended purpose	Main results for colorectal RAT
Hamilton 2013 [55]	RAT for lung, colorectal cancer in two formats: mouse mat and desk top flip chart	UK	Pre-post study	To compare referrals and investigations for colorectal and lung cancer before and after the implementation of RATs	26% increase in 2-week referrals (1173 before, 1477 after); 15% increase in colonoscopies (1762 before, 2032 after) **No conclusion possible on the effectiveness of the intervention**
Emery 2017 [56]	Education resource card including RAT for colorectal, lung and prostate cancer	Australia	Factorial cluster RCT	to measure the effect of community-based symptom awareness and GP-based educational interventions on the time to diagnosis (i.e. TDI) for patients presenting with breast, prostate, colorectal or lung cancer in rural Western Australia	**No significant differences in the median or ln mean TDI at either intervention level:** **Colorectal cancer:** -GP intervention vs control: median TDI 124 vs 122 days; ln mean difference − 0.03 95% CI − 0.51–0.45 *P* = 0.42 -community intervention vs control: median TDI 107 vs 133 days; ln mean difference − 0.26 95% CI − 0.63–0.11 *P* = 0.16;
Price 2019 [16]	Access to any RAT and/or Qcancer tool in any format	UK	Cross-sectional survey at GP practice level	To compare the mean 2WW referral rates between GP practices reporting access to RAT and/or Qcancer and those who reported no access to these tools	**No statistically significant difference between mean referral rates between practices reporting access or no access to RAT and/or Qcancer:** mean difference of 3.1 referrals per 100,000 population (95% CI − 5.5, 11.7, *p*-value 0.48)

*Abbreviations*: *ANOVA* Analysis of variance, *GP* General practitioner, *NHS* National Health Service, *OR* Odds ratio, *RAT(s)* Risk assessment tool(s), *RCT* Randomised controlled trial, *SR1* Systematic review 1, *TDI* Total diagnostic interval, *UK* United Kingdom

## Discussion

This review summarised existing evidence on development, validation, accuracy and impact of prediction models developed to help diagnosis of colorectal cancer in primary care. A large number of prediction models were identified consisting of one-off models and models from the RAT and Qcancer series. Validation and impact assessment of these models in appropriate settings is currently limited, and we found no economic evaluations of any tools.

Currently, most research on developing symptom-based colorectal cancer risk prediction models is concentrated in Europe and, in particular, the UK. Qcancer and RAT are the dominant prediction models, and highlight important knowledge gaps: the Qcancer models are developed on higher quality data (cohort data) than the RATs, and have been externally validated, but lack specific impact assessment. In contrast, the RAT models have more evidence of impact in practice, but were developed from case-control studies and have limited external validation. Ideally, this is an area for further development of the RATs, and the other models that had not been externally validated. This lack of evaluation seems consistent with prediction models in other disease areas [57].

Other systematic reviews have looked at feature-based cancer diagnostic tools in primary care. Williams and colleagues (2016) [17] conducted a systematic review of studies that described, validated or assessed the impact of colorectal cancer diagnostic tools. They identified reports on the development and/or validation of 15 models: nine relevant to primary care and six for secondary care. They also identified one study looking at referral patterns (for colorectal cancer RAT [55]). However, they did not identify any studies that tested whether patients who were diagnosed with the aid of the tool fared better than those who were diagnosed without it. In a similar review, looking at risk prediction models for screening, Usher-Smith and colleagues (2015) [53] concluded that, even though some of the colorectal cancer prediction models had potential for clinical application, there remains considerable uncertainty about their clinical utility. Similarly, Schmidt-Hansen and colleagues (2017) [58] conducted a review of lung cancer tools and found limited evidence to support the recommendation of any of the identified risk prediction tools, due to lack of external validation or cost impact assessment.

Our systematic review identified two impact studies, published after the review by Williams et al. [17], both of which indicating little evidence of an impact from using these tools in primary care. However, it is still difficult to conclude whether these tools have any impact on patient outcomes. For instance, concerns on the quality of the studies makes it unclear whether the lack of effect was due to poor implementation of the tools in practice, insufficient uptake by the GPs or limited marginal contribution of the tools in assessing the risk of cancer. The best quality study (Emery and colleagues 2017 [56]) failed to show a significant effect; however, the composite intervention used, combining older versions of several instruments (developed on populations from a different country), could have limited the effectiveness of the diagnostic tools. Thus, there is still a need for good quality studies to examine the impact of using prediction model based tools to help colorectal cancer diagnosis in primary care.

Only prediction models were included in our systematic review. Other aids, such as algorithms or guidelines may be useful, but were excluded from this review. However, the systematic review by Elias et al. [27] had a much broader inclusion criteria for “model”. The review found a previous version of the NICE guidelines to be the best performing (when validated against the CEDAR dataset). Importantly, this review did not include any of the Qcancer models, which are associated with AUCs greater than those reported for the NICE guidelines.

The systematic review followed a pre-specified protocol, and the team conducting the review are independent and experienced in systematic review methodology.

Our findings are limited by the quality of the studies included in the systematic review, in particular, among the limitations of the impact studies were lack of randomisation, lack of patient-related outcomes and use of tools on populations they were not developed for (e.g. use of a UK-developed tool on an Australian population). The outcome measures used by some of the impact studies make it difficult to interpret reports of an increase in referral rate without including reasonable assessment of the appropriateness of the referral or subsequent impact on cancer vs non-cancer diagnosis.

## Conclusion

Current evaluations provide limited evidence of the impact on patient outcomes of using feature-based cancer diagnostic tools in primary care. The lack of robust effectiveness data is also likely to be a major limiting factor in assessing their cost-effectiveness. More research is needed to externally validate prediction models that could be used as tools, as well as more research on the impact of using these tools in clinical practice. However, choice of study design and outcomes for future evaluations of the impact of tools, may not be straightforward. Practical reasons may highlight the potential need for a cluster and pragmatic trial design. Arguably, by comparing average times to diagnosis, patients not prioritised for quick referrals are less at risk of being missed. The debate, however, is ongoing on the most appropriate outcomes for evaluating interventions to improve cancer diagnosis and referral.

## Supplementary Information

**Additional file 1: Table S1.** MEDLINE literature search strategy. **Table S2a.** Development and validation studies - Characteristics (1). **Table S2b.** Development and validation studies - Characteristics (2). **Table S2c.** Development and validation studies - Model development and performance. **Table S2d.** Development and validation studies – Results. **Table S3a.** Development and validation studies - Risk of bias assessment, Questions 1 to 3. **Table S3b.** Development and validation studies - Risk of bias assessment, Questions 4 to 5. **Table S4a.** Impact Studies – Characteristics. **Table S4b.** Impact studies - Study Design. **Table S4c.** Impact studies – Results. **Table S5.** Impact studies - Critical Appraisal.

## Data Availability

The data that support the findings of this study are available within the article or its supplementary materials.

## Abbreviations

AUC: Area under the curve
CAPER: Cancer Prediction in Exeter
CEDAR: Cost-Effectiveness of a Decision rule for Abdominal complaints in Primary care
CHARMS: CHecklist for critical Appraisal and data extraction for systematic Reviews of prediction Modelling Studies
CRC: Colorectal cancer
eCDS: Electronic Cancer Decision Support
EMIS: Egton Medical Information Systems
EPOC: Effective Practice and Organisation of Care
GP(s): General practitioner(s)
NICE: National Institute for Health and Care Excellence
PRISMA: Reporting Items for Systematic Reviews and Meta-Analyses
RAT(s): Risk Assessment Tool(s)
RCT: Randomised controlled trial
TDI: Total diagnostic interval
THIN: The Health Improvement Network
UK: United Kingdom
WW: Week wait
