# Supplementary material for: Development, validation and effectiveness of diagnostic prediction tools for colorectal cancer in primary care: a systematic review
Source: BMC Cancer. 2020 Nov 10;20:1084. doi: 10.1186/s12885-020-07572-z (PMC7654186; doi:10.1186/s12885-020-07572-z)
Supplement: Supplementary file 1 — Additional file 1: Table S1. MEDLINE literature search strategy. Table S2a. Development and validation studies - Characteristics (1). Table S2b. Development and validation studies - Characteristics (2). Table S2c. Development and validation studies - Model development and performance. Table S2d. Development and validation studies – Results. Table S3a. Development and validation studies - Risk of bias assessment, Questions 1 to 3. Table S3b. Development and validation studies - Risk of bias assessment, Questions 4 to 5. Table S4a. Impact Studies – Characteristics. Table S4b. Impact studies - Study Design. Table S4c. Impact studies – Results. Table S5. Impact studies - Critical Appraisal. [file 12885_2020_7572_MOESM1_ESM.docx]

Supplementary material

Table S1. MEDLINE literature search strategy

Host: Ovid

Data Parameters: 1946 to May week 1 2017

Date Searched: 11/5/2017

Searcher: SR

Hits: 2008

| 1. exp Neoplasms/ |
| --- |
| 2. (cancer$ or neopla$).tw. |
| 3. (tumour$ or tumor$).tw. |
| 4. or/1-3 |
| 5. Primary Health Care/ |
| 6. exp General Practice/ |
| 7. General Practitioners/ |
| 8. (primary care or general practi$ or family practi$).tw. |
| 9. (primary adj3 (healthcare or health care)).tw. |
| 10. or/5-9 |
| 11. Decision Support Systems, Clinical/ |
| 12. Decision Support Techniques/ |
| 13. (tool or tools or aid$ or model or models or checklist$ or check list$ or rule or rules or algorithm$ or equation$).tw. |
| 14. or/11-13 |
| 15. "Early Detection of Cancer"/ |
| 16. (predict$ or assess$ or scor$ or risk$ or validat$ or decision$ or identif$ or diagno$ or prognos$).tw. |
| 17. (2ww or 2 week wait or two week wait or 2 week rule or two week rule).tw. |
| 18. or/15-17 |
| 19. 4 and 10 and 14 and 18 |

Table S2a. Development and validation studies - Characteristics (1)

| **Study ID** | **Prediction model** | **Cancer type(s)** | **Country** | **Setting** | **Study design** | **Stage of development** | **Data source** |
| --- | --- | --- | --- | --- | --- | --- | --- |
| **Colorectal cancer** | | | | | | | |
| Marshall 2011[29] | Bristol-Birmingham (BB) equation | Colorectal | UK | Primary care | Case-control | External validation | Derivation cohort: THIN  Validation cohort: CAPER |
| Elias 2017[27] | Bristol-Birmingham (BB) equation | Colorectal | The Netherlands | Primary care | Prospective cross-sectional | External validation | CEDAR study: Patients referred to endoscopy centres by participating Dutch primary care practices. 2009-2012 |
| Fijten 1995[28] | referred to as "Netherlands model" in Hodder 2005 below | Colorectal | The Netherlands | Primary care | Prospective cohort | Apparent performance | 290 consecutive patients with rectal bleeding presenting to 83 GPs in Limburg (Netherlands) September 1988 to April 1990 Predictors: Questionnaires completed by GPs and patients, and laboratory test results. |
| Hodder 2005[52] | Netherlands model (Fijten 1995) | Colorectal | UK | Evaluated in secondary care, model developed in primary care (Fijten and colleagues*,*  1995) | Prospective cohort | External validation | patients referred from primary care with colorectal symptoms over a 3-yr period to the Leighton Hospital, Crewe, Cheshire, UK |
| Elias 2017[27] | “Netherlands model”  (Fijten 1995) | Colorectal | The Netherlands | Primary care | Prospective cross-sectional | External validation | CEDAR study: Patients referred to endoscopy centres by participating Dutch primary care practices. 2009-2012 |
| Kop 2015[53] | No name (machine learning) | Colorectal | The Netherlands | Primary care | Retrospective case-control | Apparent performance | anonymised electronic records from two GP database systems from the Utrecht region, Netherlands, between 01-07-2006 and 31-12-2011 |
| Nørrelund 1996[31] | Danish (Nørrelund) model | Colorectal | Denmark | Primary care | Prospective cohort | Apparent performance II | Patients presenting to GPs with first episode of rectal bleeding.  Study 1: 750 GPs 1989-1991  Study 2: 450 GPs 1991-1992 |
| Elias 2017[27] | Danish (Nørrelund) model | Colorectal | The Netherlands | Primary care | Prospective cross-sectional | External validation | CEDAR study: Patients referred to endoscopy centres by participating Dutch primary care practices.  2009-2012 |
| Hippisley-Cox 2012c[22] | Qcancer | Colorectal | UK | Primary care | Open prospective cohort | internal validation II | QResearch database |
| Collins 2012[23] | Qcancer | Colorectal | UK | Primary care | Retrospective cohort | External validation | THIN database |
| Hamilton 2005[33] | RAT | Colorectal | UK | Primary care | Case-control | Apparent performance | Patients attending all 21 general practices in Exeter, Devon, UK Cases identified from the cancer registry at the Royal Devon and Exeter Hospital |
| Elias 2017[27] | RAT | Colorectal | The Netherlands | Primary care | Prospective cross-sectional | External validation | CEDAR study: Patients referred to endoscopy centres by participating Dutch primary care practices.  2009-2012 |
| Hamilton 2009[40] | RAT | Colorectal | UK | Primary care | Case-control | Model updating | THIN database |
| Stapley 2017[35] | RAT | Bowel | UK | Primary care | Case-control | Apparent performance | GPRD (currently called the CPRD) |
| **Metastatic cancer** | | | | | | | |
| Hamilton 2015[36] | RAT | Metastatic (breast, colorectal, prostate) | UK | Primary care | Case-control | Apparent performance | Patients attending 11 general practices in Devon, UK |
| **Multiple cancer sites** | | | | | | | |
| Hippisley-Cox 2013[38] | Qcancer | Females - multiple cancers (including breast, cervical, ovarian, uterine, blood, colorectal, gastro-oesophageal, lung, pancreatic, renal) | UK | Primary care | Open prospective cohort | Internal validation II | QResearch database |
| Hippisley-Cox 2013b[37] | QCancer | Males - multiple cancers (including lung, colorectal, gastro-oesophageal, pancreatic, renal tract, blood, prostate, testicular) | UK | Primary care | Open prospective cohort | Internal validation II | QResearch database |
| Muris 1995[30] | Netherlands (Muris and colleagues, 1995) model | Multiple | The Netherlands | Primary care | Prospective cohort | Apparent performance | Patients presenting to GPs for new abdominal complaints. 1989 |
| Elias 2017[27] | Netherlands (Muris and colleagues 1995) model | Colorectal, yet Muris 1995 contains multiple cancers | The Netherlands | Primary care | Prospective cross-sectional | External validation | CEDAR study: Patients referred to endoscopy centres by participating Dutch primary care practices.  2009-2012 |
| Holtedahl, 2018[39] | Prediction model for abdominal cancers | Abdominal (including all cancers of digestive organs, female genital organs, urinary organs (including testis)). Other cancers were included in additional analyses if they were associated with abdominal signs or symptoms. | Norway, Denmark, Sweden, Scotland, Belgium, Netherlands | Primary care | Prospective cohort | Apparent performance | GP records |

Abbreviations: BB = Bristol–Birmingham; BTC = biliary tract cancers; CEDAR = Cost-Effectiveness of a Decision rule for Abdominal complaints in Primary care; CPRD = Clinical Practice Research Datalink; GP(s) = general practitioner(s); GPRD = General Practice Research Database; ID = identifier; PDAC = pancreatic ductal adenocarcinoma; RAT(s) = risk assessment tool(s); SR2 = systematic review 2; THIN = The Health Improvement network; UK = United Kingdom

Table S2b. Development and validation studies - Characteristics (2)

| **Study ID** | **Participants** | **Candidate predictors** | **Outcome to be predicted** | **Sample size** | **Amount/handling of Missing data** |
| --- | --- | --- | --- | --- | --- |
| **Colorectal cancer** | | | | | |
| Marshall 2011[29] | Inclusion criteria: Cases: patients aged 30 years or older with a diagnosis of colorectal cancer between Jan 2001 and Jul 2006  Controls: seven controls per case, matched for practice, sex and age Exclusion criteria: cases with less than 2 yrs of data before diagnosis | Constipation episode, laxative prescription, diarrhoea episode, antimotility prescription, change in bowel habit (diarrhoea), change in bowel habit (constipation), change in bowel habit (no diarrhoea/ constipation), IBS diagnosis, antispasmodic prescription, rectal bleeding or melaena, faecal occult blood present, weight loss >10% in 2 yrs, weight loss 5%-10% in 2 yrs, unknown/no weight loss, abdominal pain/tenderness, abnormal rectal exam, anaemia with low Hb, iron prescription, flatulence, diabetes, BMI>30, DVT/PE, abdominal mass, mean cell volume<80fl | Diagnosis of CRC | Derivation: Cases: 5477 Controls: 38314  Discrimination based on two datasets: derivation (THIN) dataset and CAPER dataset (349 cases; 1,744 controls) | NR |
| Elias 2017[27] | Inclusion criteria: lower abdominal complaints for at least 2 wks with rectal bleeding, change in bowel habit, abdominal pain, fever, diarrhoea, weight loss, sudden onset in elderly, and/or physical examination suggestive of colorectal disease | NA. Used BB equation (Marshall 2011) | CRC | 810, including 37 with CRC | Multiple imputation |
| Fijten 1995[28] | Inclusion criteria: overt rectal bleeding reason for GP visit, or history of recent rectal blood loss (within the previous three months). Exclusion criteria: patients <18 yrs or >75 yrs, pregnant, urgent admission to a hospital (e.g., for a massive bleeding or acute abdominal pain), follow-up data not available. | Relevant items were identified from literature. Data collected by: 1. GP questionnaire after the first consultation, containing 70 variables; history, physical examination and initial management; 2. Patient questionnaire, containing 150 (somatic and psychological) questions; 3. Laboratory tests: Hb, ESR (several cutpoints), WBC and occult blood in the faeces | Diagnosis of colorectal cancer within at least 1 yr after study entry from medical records and GP. | 269 patients, 9 cancer diagnoses | Missing laboratory test data led to excluding these data in the logistic regression.  21 patients lost to follow-up. Age and gender of these patients no different to those included. |
| Hodder 2005[52] | All patients referred from primary care with colorectal symptoms from Oct 1999 to Oct 2002 | N/A. Used Fijten 1995[28] | Diagnosis of CRC | 3302 patients, 156 diagnosed with cancer | NR |
| Elias 2017[27] | Inclusion criteria: lower abdominal complaints for at least 2 wks with rectal bleeding, change in bowel habit, abdominal pain, fever, diarrhoea, weight loss, sudden onset in elderly, and/or physical examination suggestive of colorectal disease | N/A. Used Fijten 1995[28] | CRC | 810, including 37 with CRC | Multiple imputation |
| Kop 2015[53] | Patients aged ≥30 yrs Cases: a six-month period preceding the diagnosis Controls: a 6-mth period was randomly chosen between 1 Jul2006 and 31 Dec 2011 | age; gender; number of times each of 720 International Classication of Primary Care (ICPC) codes was recorded; number of times each of 86 medication (ATC) code was prescribed; number of times patient was referred to a specialist for each of the 94 referral codes; 33 attributes, each representing whether or not a patient is currently in a certain (medical) condition; number of times haemoglobin was inside/outside threshold; number of times a patient's cell volume value was inside/outside the desired range; number of times blood was found / was not found in a patient's stool; whether or not Indicates patient's medical history contains a certain temporal pattern. | Diagnostic of CRC | 808 CRC diagnoses occurring in 219.447 patients | NR |
| Nørrelund 1996[31] | Inclusion criteria: ≥40 yrs, presenting with first episode of rectal bleeding within previous 6 months. Study 1: 1989-1991, Study 2: 1991-1992  Exclusion criteria: known inflammatory bowel disease, colonic polyps, polyposis coli, colorectal cancer, predisposition to haemorrhage (e.g. coagulation defect), melaena stool.  Conducted follow-up in 1994 0 (Study 1) and 2 (Study 2) additional diagnoses of colorectal cancer observed. | Weight loss, abdominal pain, changes in bowel habits, discomfort.  Patient perceptions of cause of bleeding.  Age, gender. | CRC | Study 1: 208, including 32 with CRC  Study 2: 209, including 22 with CRC | NR |
| Elias 2017[27] | Inclusion criteria: lower abdominal complaints for at least 2 wks with rectal bleeding, change in bowel habit, abdominal pain, fever, diarrhoea, weight loss, sudden onset in elderly, and/or physical examination suggestive of colorectal disease | NA. Used Danish (Nørrelund) model | CRC | 810, including 37 with CRC | Multiple imputation |
| Hippisley-Cox 2012c[22] | Inclusion criteria: patients aged 30-84 yrs registered between 1 Jan 2000 and 30 Sept 2010;  Exclusion criteria: missing a postcode-related Townsend score; history of pancreatic cancer at baseline; red flag symptoms recorded in the 12 mths before the study entry date  Patients from GP practices using EMIS computer system for a minimum of 1 year. | Current first onset of: rectal bleeding, loss of appetite, weight-loss symptom, abdominal pain;  First onset (within last 12 mths) of: abdominal distension, constipation, diarrhoea, change in bowel habit, tiredness;  age, body mass index, alcohol status, smoking status, Townsend deprivation score (postcodes), family history of gastrointestinal cancer, previous diagnosis of cancer apart from CRC, IBD (Crohn’s disease, ulcerative colitis, coeliac disease), previous history of gastrointestinal polyp, diabetes, anaemia. | Diagnosis of colorectal cancer, defined as incident diagnosis of CRC during the 2 yrs after study | Derivation sample: 2,351,052 Validation sample: 1,236,601 | Multiple imputation to replace missing values for body mass index, alcohol and smoking status |
| Collins 2012[23] | Inclusion criteria: patients registered between 1 Jan 2000 and 30 Jun 2008, and recorded on the THIN database Exclusion criteria: prior diagnosis of CRC, patients registered <12 mths with the general practice, had invalid dates, >30 yrs old, >85 yrs old. | NA. Used Qcancer (colorectal), see Hippisley-Cox 2012c | Diagnosis of colorectal cancer, Defined as incident diagnosis of CRC during the 2 yrs after study entry | 2,135,540 patients, including 3,712 cases of CRC (1,676 women and 2,036 men) | Multiple imputation using all predictors plus the outcome variable was used to replace missing values for alcohol consumption |
| Hamilton 2005[33] | Inclusion criteria: Cases: patients aged 40 yrs or over with a primary CRC, diagnosed from 1998 to 2002 from one hospital. Controls: five for each case matched on sex, general practice, and age Exclusion criteria (both): unobtainable records; no consultations in the 2 yrs before diagnosis; previous colorectal cancer; or residence outside Exeter at the time of diagnosis | All related features (symptoms and variables) were investigated only features occurring in at least 2.5% of either cases or controls were analysed | Diagnosis of CRC within 2 yrs from presentation | 349 cases 1,744 controls | NR |
| Elias 2017[27] | Inclusion criteria: lower abdominal complaints for at least 2 wks with rectal bleeding, change in bowel habit, abdominal pain, fever, diarrhoea, weight loss, sudden onset in elderly, and/or physical examination suggestive of colorectal disease | NA. Used RAT (colorectal) | CRC | 810, including 37 with CRC | Multiple imputation |
| Hamilton 2009[40] | Inclusion criteria: cases: all patients with colorectal cancer, aged 30 years or older, diagnosed between January 2001 and July 2006 controls: up to seven for each case, matched for practice, sex, and age Exclusion criteria: less than 2 years of data recorded prior to the diagnosis | 23 candidate variables (features) were identified from a review of the literature | Diagnosis of CRC within 2 yrs from presentation | 5,477 cases, 38,314 controls | NR |
| Stapley 2017[35] | Inclusion criteria: Cases: patients with IBD and CRC, aged 18- 49 yrs at diagnosis, between Jan 2000 and Dec 2013 Controls: 3 controls were matched on sex, general practice, and to 1 yr of age of the case.  Exclusion criteria: Cases and controls with no consultations in the year before the index date; controls that had a previous diagnostic code of CRC/IBD, diagnosis before 2000. | All symptoms, physical signs or abnormal investigations related to CRC/IBD, that occurred in ≥5% of cases or controls were retained | Diagnosis of either IBD and CRC within one year from presentation with symptoms | CRC: 1,661 cases, 3,979 controls IBD: 9,578 cases, 22 947 controls | NR |
| **Metastatic cancer** | | | | | |
| Hamilton 2015[36] | Inclusion criteria: cases: deceased with a prior record of breast, colorectal, or prostate cancer and radiologically or histologically proven metastatic cancer. controls: 2 controls per case, alive at the time of the diagnosis of metastatic cancer in the case, matched for practice, sex, age; one with same (non-metastatic) cancer, one without cancer Exclusion criteria: primary cancer considered incurable at the time of initial diagnosis, or metastatic spread had occurred within 6 mths of diagnosis of the primary cancer; cases in which the primary cancer was diagnosed before 40 yrs of age; patients whose metastases occurred before registration at the current practice; cases, the full record for which had been archived off site after death. | NR. 207 separate ‘features’ were identified in at least >2% of cases. | Diagnosis of metastatic cancer within 6 mths from diagnosis of primary cancer | Cases: 162  Cancer controls: 152  Non-cancer controls: 145 | NR |
| **Multiple cancer sites** | | | | | |
| Hippisley-Cox 2013[38] | Inclusion criteria: Females aged 25–89 yrs registered with participating practices between 1 Jan 2000 and 1 Apr 2012;  Exclusion criteria: Missing a postcode-related Townsend score; red flag symptoms recorded in the 12 months before the study entry date.  Patients from GP practices using EMIS computer system for a minimum of 1 yr. | Red flag symptoms and more general symptoms, plus risk factors:  Age, BMI, smoking status, alcohol use, Townsend deprivation score (postcodes), previous diagnosis of cancer, anaemia, family history of breast cancer, family history of gastrointestinal cancer, family history of ovarian cancer, benign breast disease, chronic pancreatitis, type 1 diabetes, type 2 diabetes, endometriosis, endometrial hyperplasia or polyp, fibroid, polycystic ovarian disease, rheumatoid arthritis, systemic lupus erythematosis, HIV or AIDS, oral contraceptive use, hormone replacement therapy. | Diagnosis of cancer within 2 yrs after study entry | Derivation sample: 1,240,864 Validation sample: 667,603 | Multiple imputation was used in the validation cohort to replace missing values for BMI, alcohol, and smoking |
| Hippisley-Cox 2013b[37] | Inclusion criteria: males aged 25–89 yrs registered with participating practices between 1 Jan 2000 and 1 Apr 2012;  Exclusion criteria: missing a postcode-related Townsend score; red flag symptoms recorded in the 12 mths before the study entry date.  Patients from GP practices using EMIS computer system for a minimum of 1 yr. | Red flag symptoms and more general symptoms, plus risk factors:  age, BMI, smoking status, alcohol use, Townsend deprivation score (postcodes), previous diagnosis of cancer, anaemia, family history of gastrointestinal cancer, family history of prostate cancer, chronic pancreatitis, type 1 diabetes, type 2 diabetes. | Diagnosis of cancer within 2 yrs after study entry | Derivation sample: 1,263,071 Validation sample: 679,174 | Multiple imputation was used in the validation cohort to replace missing values for BMI, alcohol, and smoking |
| Muris 1995[30] | Inclusion criteria: 18-75 yrs, consulting GP for new abdominal complaints lasting at least 2 wks, consenting to participate. | Age and sex. WBC count , erythrocyte sedimentation rate, low Hb level, positive faecal occult blood test. Low somatization score, no depression, high self-esteem, social inadequacy, plus 23 symptoms. | Multiple cancers | 933, including ~18 having cancer | NR |
| Elias 2017[27] | Inclusion criteria: lower abdominal complaints for at least 2 wks with rectal bleeding, change in bowel habit, abdominal pain, fever, diarrhoea, weight loss, sudden onset in elderly, and/or physical examination suggestive of colorectal disease | NA. Used Muris 1995[30] | CRC, yet Muris 1995[30] is multiple cancers | 810, including 37 with CRC | NR |
| Holtedahl, 2018[39] | 493 GPs recruited via Cancer and Primary Care Research International Network, Feb-July 2011.  GPs recorded consultations over a period of 10 days for patients ≥ 16 years old. If abdominal symptoms were mentioned in the consultation, specific symptom-related questions were asked using a proforma.  GPs also identified all those with a diagnosis of abdominal cancer within 6 months after the GP survey regardless of whether patient attended during the survey period. | Abdominal pain (lower and upper), constipation, diarrhoea, distended abdomen/bloating, increased belching/flatulence, acid regurgitation, rectal bleeding, unexpected genital bleeding, macroscopic haematuria, increased urinary frequency, other abdominal problems | Cancer diagnosis within 180 of GP survey. Diagnosis taken from GP records 8 months after GP survey. Study authors distinguished between abdominal and non-abdominal cancers | 61802 patients, including 175 cases (0.28%) | NR |

Notes: AIDS = acquired immune deficiency syndrome; BB = Bristol–Birmingham; BMI = body mass index; BTC = biliary tract cancers; DVT = deep venous thrombosis; EMIS = Egton Medical information Systems; ESR = erythrocyte sedimentation rate; GP(s) = general practitioner(s); GPRD = General Practice Research Database; Hb = haemoglobin; HIV = human immunodeficiency virus; IBD = irritable bowel disease; IBS, irritable bowel syndrome; ID = identifier; mth(s) = month(s); NA = not applicable; NR = not reported; PDAC = pancreatic ductal adenocarcinoma; PE = pulmonary embolism; SR2 = systematic review 2; THIN = The Health Improvement Network; WBC = white blood cell; wk(s) = week(s); yr(s) = year(s)

Table S2c. Development and validation studies - Model development and performance

| **Study ID** | **Model development** | **Model performance** |
| --- | --- | --- |
| **Colorectal cancer** | | |
| Marshall 2011[29] | Multivariable conditional logistic regression analysis. Initial univariable conditional logistic regression analysis was carried out with the initial predictor variables and some variables were combined. Variables associated with colorectal cancer with a P-value <0.1 were entered into multivariable conditional logistic regression. | ROC curves, sensitivity, LRs and PPVs also reported.  In the CAPER dataset, AUCs: BB 0.92 (95% CI 0.91 to 0.94); CAPER 0.91 (95% CI 0.89 to 0.93); NICE guidelines 0.75 (95% CI 0.72 to 0.79).  Likelihood ratios:  NICE 1: 5.7, NICE 2: 13.3, NICE 3: 12.5, CAPER: 13.4, BB: 14.7 |
| Elias 2017[27] | NA. Used BB equation[29] | NPV (95% CI): 100 (98, 100)  PPV (95% CI): 7 (5, 9)  Sensitivity (95% CI): 97 (85, 100)  Specificity (95% CI): 36 (32, 40)  AUC (95% CI): 0.84 (0.77, 0.90) |
| Fijten 1995[28] | Forward stepwise logistic regression analysis. Variables showing an association (P-value < 0.1) with cancer were included in the multivariate model. | Diagnostic index: AUC 0.97 (no estimate of variance reported). Also reported sensitivity, specificity, predictive values, ORs.  Diagnostic index + presence of polyps: AUC 0.92  For both, calculated cut-offs to maximise sensitivity and specificity. |
| Hodder 2005[52] | Used Fijten 1995[28] risk prediction model.  Also assessed performance of guidelines and scores that were not based on risk prediction models (results not reported here) | AUC of 0.775 (SE 0.02) |
| Elias 2017[27] | NA. Used Fijten 1995[28] | NPV (95% CI): 99 (95, 1000)  PPV (95% CI): 10 (7, 14)  Sensitivity (95% CI): 98 (83, 100)  Specificity (95% CI): 29 (24, 34)  AUC (95% CI): 0.72 (0.62, 0.81) |
| Kop 2015[53] | Models were generates using logistic regression, RF, SVM and the CART algorithm. | A priori algorithm: the records are scanned first to create frequent patterns of size 1, 1 -patterns. These patterns are used to generate successively larger patterns, i.e. k-patterns are used to obtain frequent k+1-patterns. Generating a k+1-pattern is, however, more elaborate than for standard a priori, because the generated candidate patterns need to accommodate for multiple possible (s) and (c) relations. This results in more k+1-patterns being tested for frequency than with standard a priori, increasing the complexity |
| Hippisley-Cox 2012c[22] | Cox proportional hazards. Rubin’s rules were used to combine the results across the imputed datasets.  Fractional polynomials used for non-linear risk relationships. Fitted full model, variables retained if HR <0.80, HR >1.20 and P-value 0.01. | Females R^2^ (%): 64.8 (63.2 to 66.3) D statistic: 2.78 (2.68 to 2.87) AUC: 0.89 (0.88 to 0.90) Males R^2^ (%): 66.7 (65.3 to 68.0) D statistic: 2.90 (2.81 to 2.98) AUC: 0.906 (0.899 to 0.913) |
| Collins 2012[23] | NA. Used Qcancer (colorectal), see Hippisley-Cox 2012c[22] | Men  Multiple imputation (m=10) (n=1 059 765) R^2^ (95% CI) 68.32 (67.32–69.32) D-statistic (95% CI) 3.00 (2.93–3.07) c-Statistic (95% CI) 0.918 (0.913–0.923) Complete-case (n=417 560) R^2^ (95% CI) 65.30 (63.71–66.89) D-statistic (95% CI) 2.81 (2.71–2.91) c-Statistic (95% CI) 0.901 (0.892–0.910)  Women  Complete-case (n=1 075 775) R^2^ (95% CI) 65.81 (64.62–67.01) D-statistic (95% CI) 2.84 (2.76–2.92) c-Statistic (95% CI) 0.909 (0.903–0.915) |
| Nørrelund 1996[31] | Mann-Whitney and Chi-squared tests, and logistic regression. No further details reported. | Study 1 (apparent performance I)  age>69: Sensitivity 75%, Specificity 76%, PPV 36%, NPV 94%  age>69 plus change in bowel habits: Sensitivity 44%, Specificity 94%, PPV 56%, NPV 90%  change in bowel habits plus patient belief bleeding is due to cancer: Sensitivity 22%, Specificity 97%, PPV 58%, NPV 87%  Study 2 (apparent performance II) New bleeders  age>69: Sensitivity 46%, Specificity 72%, PPV 18%, NPV 91%  age>69 plus change in bowel habits: Sensitivity 15%, Specificity 88%, PPV 13%, NPV 85%  change in bowel habits plus patient belief bleeding is due to cancer: Sensitivity 0%, Specificity 95%, PPV 0%, NPV 87%  Study 2 (apparent performance II) New or changed bleeders  age>69: Sensitivity 45%, Specificity 75%, PPV 23%, NPV 91%  age>69 plus change in bowel habits: Sensitivity 23%, Specificity 88%, PPV 24%, NPV 87%  change in bowel habits plus patient belief bleeding is due to cancer: Sensitivity 5%, Specificity 96%, PPV 14%, NPV 86% |
| Elias 2017[27] | NA. Used Danish (Nørrelund) model | NPV (95% CI): 93 (71, 99)  PPV (95% CI): 8 (6, 12)  Sensitivity (95% CI): 95 (80, 99)  Specificity (95% CI): 6 (3, 9)  AUC (95% CI): 0.60 (0.48, 0.72) |
| Hamilton 2005[33] | Variables associated with cancer in univariable analyses, using a P-value of 0.1 or less, entered the multivariable analysis In first stage, similar variables were grouped together; in second stage analyses were repeated with the new groups | 10 features (constipation, diarrhoea, rectal bleeding, loss of weight, abdominal pain, abdominal tenderness, abnormal rectal exam, anaemia (Hb 10 − 13 g/dl; Hb <10 g/dl), blood sugar >10 mmol/l) were associated with CRC before diagnosis. The PPVs (95% CI) of these were rectal bleeding 2.4% (1.9, 3.2); weight loss 1.2% (0.91, 1.6); abdominal pain 1.1% (0.86, 1.3); diarrhoea 0.94% (0.73, 1.1); constipation 0.42% (0.34, 0.52); abnormal rectal examination 4.0% (2.4, 7.4); abdominal tenderness 1.1% (0.77, 1.5); Hb <10.0 g/dl 2.3% (1.6, 3.1); positive faecal occult bloods 7.1% (5.1, 10); blood glucose410 mmol/l 0.78% (0.51, 1.1): all P<0.001.  See Figure 2 of the publication for additional PPVs.  There was an interaction (P<0.016) between haemoglobin  subcategory and age group, whereby the association between  anaemia and cancer was stronger among younger patients. To  illustrate this, the likelihood ratios (95% confidence interval (CI))  for the younger age group (aged 40–69) were haemoglobin 12.9–  12.0 g dl1, 15 (6.8, 33), haemoglobin 11.9–10.0 g dl1, 17 (10, 29) and haemoglobin o10 g dl1, 13 (6.7, 27). For patients aged over 70 years the likelihood ratios were 3.1 (1.7, 5.5), 2.7 (1.8, 3.9), and 8.9 (6.4, 12), respectively. |
| Elias 2017[27] | NA. Used RAT (colorectal) model[33] | NPV (95% CI): 99 (98, 100)  PPV (95% CI): 8 (6, 10)  Sensitivity (95% CI): 95 (82, 99)  Specificity (95% CI): 45 (41, 49)  AUC (95% CI): 0.81 (0.75, 0.88) |
| Hamilton 2009[40] | variables with a univariable association with cancer significant with a P value < 0.1 were entered into a staged multivariable analysis | PPVs were estimated  Six symptoms and two abnormal investigations (anaemia and microcytosis) were independently associated with colorectal cancer. The positive predictive values of symptoms were: rectal bleeding, positive predictive value for a male aged ≥ 80 years 4.5% (95% confidence interval 3.5, 5.9); change in bowel habit 3.9% (2.8, 5.5); weight loss 0.8% (0.5, 1.3); abdominal pain 1.2% (1.0, 1.4); diarrhoea 1.2% (1.0, 1.5) and constipation 0.7% (0.6, 0.8). Positive predictive values were lower in females and younger patients. Only 27% of patients had reported either of the two higher risk symptoms.  Likelihood ratios (95% CI):  Constipation 2.6 (2.4 to 2.7),  Diarrhoea 3.2 (3.0 to 3.4)  Change in bowel habit 5.5 (5.2 to 5.8)  Rectal bleeding 6.0 (5.7 to 6.3)  5.0–9.9% Weight loss 1.6 (1.4 to 1.8)  ≥ 10% weight loss 2.9 (2.6 to 3.1)  Abdominal pain 3.5 (3.3 to 3.7)  Haemoglobin < 12.0 g/dl 4.4 (4.2 to 4.6)  Mean red cell volume < 80 fl 2.8 (2.4 to 3.1)  Irritable bowel syndrome 2.4 (2.1 to 2.8)  Diabetes 1.2 (1.1 to 1.3)  Obesity 1.0 (0.93 to 1.1) |
| Stapley 2017[35] | conditional logistic regression variables independently associated with pancreatic cancer with a P-value <0.1 were entered into the multivariable analysis multivariable analysis performed in three stages; final model used a threshold of P<0.02 | PPVs were derived, using national incidence data to estimate prior odds (see Figure 2 in publication).  Likelihood ratios (95% CI):  Diarrhoea 13.8 (12.6 to 15)  Abdominal pain 4.8 (4.5 to 5)  Rectal bleeding 31.6 (27.5 to 36.5)  Change in bowel habit 26.9 (20.9 to 34.6)  Raised inflammatory markers 13 (11.9 to 14.2)  Low haemoglobin 7.6 (6.9 to 8.3)  Raised platelets 19.5 (16.9 to 22.5)  Raised white cell count 7.2 (6.5 to 8)  Abnormal liver function 3.3 (3 to 3.5)  Low mean red cell volume 9.1 (8 to 10.3) |
| **Metastatic cancer** | | |
| Hamilton 2015[36] | Conditional logistic regression Univariable analyses were performed initially, retaining variables with a P-value of <0.1 to enter into multivariable analyses; only variables that were present in >2% of the cases were studied cancer controls and healthy controls were used in separate analyses, and the cancer sites were analysed separately and also merged and a unified analysis performed clinically plausible interaction terms were added to each model, and likelihood ratio testing was applied to test whether they improved the models | NR |
| **Multiple cancer sites** | | |
| Hippisley-Cox 2013[38] | Multinomial logistic regression was used to estimate the coefficients for each predictor variable for each type of cancer. Fitted full model and variables retained if P<=0.01. Fractional polynomials were used to model non-linear risk relationships with continuous variables. | Discrimination [Table 6]: All ROC statistic values for each cancer type were above 0.79 except for cervix (0.73). The highest ROC values were for lung cancer (0.91) and uterine cancer (0.91). Calibration [Figure 1]: Compared mean predicted ad observed risks. Overall, the model was well calibrated for each cancer type except for ‘other cancer’ which showed a degree of over prediction. Classification measures [Table 7]: Symptoms with the highest positive predictive values for any cancer (regardless of type) were: breast lump (11%), haemoptysis (8%), dysphagia (8%), and post-menopausal bleeding (7%). The positive predictive value for anaemia was 6% and for venous thrombo-embolism was 5%.  Used validation cohort to define thresholds for 1%, 5% and 10% risk. Calculated sensitivity, specificity, NPV and PPV at these thresholds. |
| Hippisley-Cox 2013b[37] | Multinomial logistic regression was used to estimate the coefficients for each predictor variable for each type of cancer. Fitted full model and variables retained if P<=0.01. Fractional polynomials were used to model non-linear risk relationships with continuous variables. | Discrimination [Table 6]: All ROC statistic values for each cancer type were above 0.82 indicating very good discrimination. The highest ROC values were for renal tract cancer (0.94), gastro-oesophageal cancer (0.93), and lung cancer (0.92). The lowest was for testicular cancer (0.82). Calibration [Figure 1]: Compared mean predicted ad observed risks. Overall, the model was well calibrated for each cancer type except for the ‘other cancer’ model which showed a degree of over prediction. Classification measures [Table 7]: Symptoms with the highest positive predictive values for any cancer (regardless of type) were anaemia (19%), urinary retention (14%), dysphagia (13%), haematuria (13%), weight loss (11%), neck lump (10%), haemoptysis (10%). The positive predictive value for venous thrombo-embolism was 6%. The sensitivity of single symptoms was generally low with the highest value being 16% for abdominal pain.  Used validation cohort to define thresholds for 1%, 5% and 10% risk. Calculated sensitivity, specificity, NPV and PPV at these thresholds. |
| Muris 1995[30] | Variables where P<0.25 in univariate analyses were entered into multiple stepwise forward logistic regression. | NR |
| Elias 2017[27] | NA. Used Muris model[30] | NPV (95% CI): 99 (97, 100)  PPV (95% CI): 6 (4, 8)  Sensitivity (95% CI): 97 (86, 100)  Specificity (95% CI): 28 (25, 31)  AUC (95% CI): 0.62 (0.54, 0.70) |
| Holtedahl, 2018[39] | Cox proportional hazards models. Report univariate and multivariate analyses (for the most frequent symptoms and combinations of symptoms adjusted for sex).  Authors report no evidence to reject proportional hazards assumption.  Main analyses included all patients with new abdominal cancer diagnosis within 180 days. Other analyses looked at all cancers, or abdominal cancers also beyond 180 days.  0.05 level of statistical significance used. | Sensitivity, specificity, LRs, and PPVs reported for individual symptoms and combinations of symptoms (by age group or gender)  Likelihood ratios (95% CIs)  Abdominal pain, upper part 5.3 (3.8 to 7.4)  Abdominal pain, lower part 3.4 (2.2 to 5.1)  Constipation 8.2 (5.1 to 13.2)  Diarrhoea 2.5 (1.3 to 5.0)  Distended abdomen, bloating 5.9 (3.7 to 9.3)  Increased belching, flatulence 6.4 (3.4 to 12.2)  Acid regurgitation 5.2 (2.9 to 9.6)  Rectal bleeding 13.6 (8.3 to 22.3)  Unexpected genital bleeding 5.4 (1.7 to 16.7)  Macroscopic haematuria 15.7 (6.8 to 34.5)  Increased urinary frequency 4.8 (2.6 to 8.8)  Other abdominal problems 5.6 (3.6 to 8.8)  >1 abdominal symptom 5 (3.8 to 6.6)  At least 1 abdominal symptom 4.4 (3.7 to 5.2)  Lack of appetite 7.4 (4.7 to 11.5)  Unusual tiredness 7.3 (4.6 to 11.6)  Involuntary weight loss 12.7 (7.1 to 22.7)  >1 non-specific symptom 11.6 (6.8 to 19.7)  Any non-specific symptom 6.8 (4.8 to 9.5) |

Abbreviations: AUC = area under the curve; BB = Bristol–Birmingham; CAPER = Cancer Prediction in Exeter; CART = classification and regression tree; CI = confidence interval; CRC = colorectal; E = expected; Hb = haemoglobin; HR = hazard ratio; mth(s) = month(s); NA = not applicable; NICE = National Institute for Health and Care Excellence; NPV = negative predictive values; NR = not reported; O= observed; OR = odds ratio; PPV = positive predictive value; RAT(s) = risk assessment tool(s); RF = random forest; ROC = receiver operating characteristic; SE = standard error; SR2 = systematic review 2; SVM = support vector machine

Note: Table and figure references in the above table are those relevant to the source publication the data are extracted from

Table S2d. Development and validation studies - Results

| **Study ID** | **Model evaluation** | **Results** | **Interpretation and discussion** |
| --- | --- | --- | --- |
| **Colorectal cancer** | | | |
| Marshall 2011[29] | Apparent performance with THIN dataset  BB validated with CAPER dataset | Model predictors and ORs (95% CIs): constipation 2.06 (1.88, 2.26), diarrhoea 2.38 (2.14, 2.66), change in bowel habit 13.83 (11.70, 16.34), abdominal pain 3.82 (3.49, 4.18), rectal bleeding 20.11 (17.35, 23.32), Hb 13-13.99g/dl 1.33 (1.18, 1.50), Hb 12-12.99g/dl 1.63 (1.42, 1.87), Hb 11-11.99g/dl 2.54 (2.16, 2.99), Hb 10-10.99g/dl 5.18 (4.19, 6.39), Hb 9-9.99g/dl 8.08 (6.13, 10.65), Hb <9g/dl 15.94 (11.78, 21.57), mean cell vol 80-84.99fl 2.71 (2.30, 3.19), mean cell vol <80fl 7.67 (6.23, 9.44), weight loss =>10% 2.92 (2.39, 3.57), weight loss 5%-10% 1.37 (1.09, 1.73) | Both multivariable BB and CAPER equations performed significantly better than NICE referral guidelines. |
| Elias 2017[27] | External validation | Elias ranked the BB equation as 6^th^ out of 19 models evaluated. | The top ranked model were NICE guidelines. Note that Elias used a very broad definition of prediction model which included guidelines and weighted scores. |
| Fijten 1995[28] | Development dataset only | Diagnostic index = -6.7 + 2.1 ((age-50)/10) + 2.3 if change in bowel habit + 2.1 if blood mixed with or on stool. (Hosmer Lemeshow P = 0.507), log likelihood = -17.9).  Cut-off for maximising sensitivity (100%) and specificity (90%) was 0.042.  Diagnostic index with presence of polyps: -4.8 + 1.4 ((age-50)/10) + 1.9 if change in bowel habit + 2.1 if blood mixed with or on stool. (Hosmer Lemeshow P = 0.49), log likelihood = -32.4).  Cut-off for maximising sensitivity and specificity was 0.058. | The combination of age, change in bowel habit and blood seen mixed with or on stool can serve as a useful diagnostic tool for the prediction of CRC carcinoma (and overtly bleeding polyps) |
| Hodder 2005[52] | External validation | The Netherlands model had better discrimination compared to the Harvard model, but inferior to the WNS | |
| Elias 2017[27] | External validation | CEDAR dataset included participants older than those in original Fijten 1995[28] derivation dataset. Based on the results, Elias ranked the Fijten 1995[28] model as 13 out of 19 models. | The top ranked model were NICE guidelines. Note that Elias used a very broad definition of prediction model which included guidelines and weighted scores. |
| Kop 2015[53] | Five subsets were selected and four machine learning algorithms applied to them in a 5-fold cross validation fashion: non-temporal (941 non-temporal attributes + age/gender); temporal (n temporal patterns + age/gender); all (941 + n + age/gender); knowledge-driven (31 attributes as described in Marshall 2011 + age/gender); age/gender (benchmark; age and gender only). | AUCs and 95% CIs for subsets: non-temporal: LR 0.792 (0:771 - 0:813); RF 0.883 (0:866 - 0:900); SVM 0.804 (0:784 - 0:824); CART 0.819 (0:799 - 0:839) temporal: LR 0.893 (0:877 - 0:909); RF 0.882 (0:865 - 0:899); SVM 0.861 (0:843 - 0:879); CART 0.863 (0:845 - 0:881) all: LR 0.796 (0:775 - 0:817); RF 0.881 (0:864 - 0:898); SVM 0.832 (0:813 - 0:851); CART 0.818 (0:798 - 0:838) knowledge-driven: LR 0.854 (0:836 - 0:872); RF 0.896 (0:880 - 0:912); SVM 0.867 (0:849 - 0:885); CART 0.860 (0:842 - 0:878) age/gender only: LR 0.844 (0:825 - 0:863); RF 0.838 (0:819 - 0:857); SVM 0.862 (0:844 - 0:880); CART 0.828 (0:808 - 0:848) | study suggests metabolic syndrome as potential predictor |
| Nørrelund 1996[31] | Validated in Study 2 | Only age was found to be a statistically significant predictor of cancer from Study 1:  Age 70-79yrs adj OR 9.26 (95%CI 3.32, 25.82); age 80yrs+ adj OR 9.90 (95%CI 2.03, 48.36).  No statistically significant variables were found in Study 2 to predict cancer. | Reported symptoms of weight loss, abdominal pain, change in bowel habits or discomfort were not found to be predictors of CRC cancer in either Study 1 or 2. |
| Elias 2017[27] | External validation | Elias ranked the Danish (Nørrelund) model as 18^th^ out of 19 models evaluated. | The top ranked model were NICE guidelines. Note that Elias used a very broad definition of prediction model which included guidelines and weighted scores. |
| Hippisley-Cox 2012c[22] | Split sample validation (66%/33%) | Predictors and (fully adjusted) HR (95% CI)  Females: family history of GI cancer 1.39 (1.02, 1.89), Hb <11g/dl 3.26 (2.84, 3.74), current rectal bleeding 32.3 (27.7, 37.6), current abdominal pain 6.90 (5.91, 8.06), current appetite loss 2.43 (1.70, 3.47), current weight loss 7.70 (5.32, 11.1)  Males: trivial drinker 1.07 (0.95, 1.20), light drinker 1.20 (1.06, 1.35), moderate/heavy drinker 1.43 (1.25, 1.63), family history of GI cancer 1.52 (1.12, 2.07), Hb <11g/dl 3.33 (2.86, 3.87), current rectal bleeding 27.0 (23.5, 31.1), current abdominal pain 6.78 (5.76, 7.97), current appetite loss 2.15 (1.53, 3.03), current weight loss 4.07 (3.42, 4.85), 2.25 (1.47, 3.46). | State that the algorithm performed well, with good discrimination and calibration |
| Collins 2012[23] | External validation with a different cohort | Model calibration is very good with close agreement between predicted and observed CRC cancer risks across all tenths of risk. | |
| Hamilton 2005[33] | Development dataset only | ORs (95% CIs):  Rectal bleeding 15 (9.0, 2.4), weight loss 2.7 (1.7, 4.6), number of episodes of abdominal pain 2.2 (1.7, 2.8), constipation 2.0 (1.2, 3.3), number of episodes of diarrhoea 1.6 (1.3, 2.0), rectal disease on examination 13 (4.7, 37), tenderness on palpitation of abdomen 3.6 (1.7, 7.8), positive FOB 81 (20, 330), Hb 12.0-12.9g/dl 2.5 (0.95, 6.8), Hb 10.0-11.9 g/dl 4.3 (2.1, 9.0), Hb <10g/dl 13 (6.2, 28), blood sugar >10mmol/l 2.0 (1.3, 3.1).  Interaction terms: abdominal pain with tenderness 0.56 (0.38, 0.82), positive FOB with Hb <10g/dl 0.020 (0.0015, 0.27). | 10 symptoms, signs or investigation results were independently associated with CRC cancer. Five of these remained associated with cancer 180 days before diagnosis. |
| Elias 2017[27] | External validation | Elias ranked the RAT (CRC) model as 10^th^ out of 19 models evaluated. The top ranked model were NICE guidelines. | Note that Elias used a very broad definition of prediction model which included guidelines and weighted scores. |
| Hamilton 2009[40] | Development dataset only | ORs (95% CIs)  Rectal bleeding 20 (17, 23), change in bowel habit 14 (12, 17), abdominal pain 3.9 (3.6, 4.3), diarrhoea 2.4 (2.1, 2.7), constipation 2.1 (1.9, 2.3), weight loss 5.0-9.9% 1.2 (0.99, 1.5), weight loss =>10% 2.5 (2.1, 3.0), Hb 12.0-12.9 g/dl 1.7 (1.5, 1.9), Hb 11.0-11.9 g/dl 2.8 (2.4, 3.2), Hb 10.0-10.9 g/dl 5.9 (4.8, 7.2), Hb 9.0-9.9 g/dl 9.3 (7.1, 12), Hb <9 g/dl 18 (14, 25), mean red cell volume < 80fl 6.5 (5.3, 7.9). | there is a need to improve identification of CRC cancer among the large number of patients presenting only with low-risk symptoms |
| Stapley 2017[35] | Development dataset only | OR (95% CIs)) for CRC:  Diarrhoea 7.7 (4.3, 14), abdominal pain 6.0 (4.2, 8.7), rectal bleeding 54 (26, 110), change in bowel habit 58 (21, 160), constipation 7.9 (4.3, 14), nausea/vomiting 2.7 (1.4, 5.1), rectal mass 190 (51, 720), raised inflammatory markers 3.1 (2.0, 4.7), low Hb 5.2 (3.2, 8.5), low mean red cell volume 4.3 (2.3, 8.0). | Rectal bleeding and change in bowel habit are strongly predictive of CRC/IBD when combined with abnormal haematology. |
| **Metastatic cancer** | | | |
| Hamilton 2015[36] | Development dataset only | Adjusted OR (95% CI)  Vs Cancer controls: groin pain 10.2 (1.2, 8.2), pleurisy/pleural effusion 10.2 (1.1, 9.2), shoulder pain 5.3 (1.6, 1.8), loss of appetite 4.0 (1.2, 1.3), vomiting 3.5 (1.3, 9.4), low back pain 2.5 (1.1, 5.6), abnormal liver function 3.5 (1.6, 7.5)  Vs Healthy controls: vomiting 3.6 (1.3, 1.0), 4.2 (1.5, 1.2), flank/loin pain 19.4 (1.8, 2.10), chest pain musculoskeletal 5.3 (1.7, 1.6), oedema3.4 (1.1, 10), abnormal liver function 5.1 (1.9, 1.4) | The scarcity of specific symptoms and the fairly common occurrence of non-specific symptoms (vomiting and loss of appetite) may explain delays in the diagnosis of metastases |
| **Multiple cancer sites** | | | |
| Hippisley-Cox 2013[38] | Split sample validation (66%/33%) | Each cancer model contained the following number of predictors (see additional tables on Qcancer.org for details):  Breast 10, cervical 12, ovarian 11, uterine 7, blood 10, CRC 13, gastro-oesophageal 13, lung 15, pancreatic 14, renal 11, other cancers 22. | A new algorithm designed to estimate the absolute risk of having existing but as yet undiagnosed cancer was developed and validated |
| Hippisley-Cox 2013b[37] | Split sample validation (66%/33%) | Each cancer model contained the following number of predictors (see additional tables on Qcancer.org for details):  Prostate 14, testicular 3, blood 14, CRC 12, gastro-oesophageal 13, lung 17, pancreatic 15, renal tract 8, other cancers 20. | A new algorithm designed to estimate the absolute risk of having existing but as yet undiagnosed cancer was developed and validated |
| Muris 1995[30]1 | Apparent performance | Statistically significant predictors (adjusted ORs (95% CI)) were:  No specific character to pain 5.70 (1.97, 16.51)  Weight loss 4.36 (1.72, 11.11)  ESR >20mm/hour 3.00 (1.10,8.17)  Male sex 2.37 (1.20, 6.99)  Greater age (yrs) 1.08 (1.04, 1.11) | The aim of the study was to identify predictors for organic disease, with a secondary analysis looking at predictors for neoplasms. |
| Elias 2017[27] | External validation | Elias ranked the Muris[30] model as 13^th^ out of 19 models evaluated. | The top ranked model were NICE guidelines. Note that Elias used a very broad definition of prediction model which included guidelines and weighted scores. |
| Holtedahl, 2018[39] | Developmental dataset only | HR (95% CI)  Abdominal pain (upper, single symptom) 4.8 (1.9, 11.8)  Abdominal pain (lower, single symptom) 5.8 (2.4, 14.3)  Constipation (single symptom) 6.8 (2.1, 21.8)  Rectal bleeding (single symptom) 19.1 (8.7, 41.7)  Any other single symptoms (grouped) 4.7 (2.8, 7.9)  2 abdominal symptoms 4.6 (2.5, 8.5)  ≥3 abdominal symptoms 14.0 (9.1, 21.6)  Also reported HRs (95%CIs) for combinations of symptoms – see Table 4 of Holtedahl. | The strength of the association between abdominal symptoms and cancers highlights the importance of responding to these symptoms. But as some new cancers did not involve these symptoms, clinical suspicion is needed. |

Abbreviations: adj = adjusted; AL = acute leukaemia; AUC, area under curve; BB = Bristol–Birmingham; BC = breast cancer; BTC = biliary tract cancer; CAPER = Cancer Prediction in Exeter; CART = classification and regression trees; CEDAR = Cost-Effectiveness of a Decision rule for Abdominal complaints in Primary care; CI = confidence interval; CL = chronic leukaemia; CRC = colorectal cancer; ESR = erythrocyte sedimentation rate; FOB = faecal occult blood; GI = gastrointestinal; GP(s) = general practitioner(s); Hb = haemoglobin; IBD = irritable bowel disease; LR, likelihood ratio; mth(s) = month(s); NICE = National Institute of Health and Care Excellence;OR = odds ratio; PDAC = pancreatic ductal adenocarcinoma; PPV, positive predictive value; RF = random forest; ROC, receiver operating characteristic; SVM = support vector machine; THIN = The Health Improvement Network; WBC = white blood cell; WNS = weighted numerical score; yr(s) = year(s)

Table S3a. Development and validation studies - Risk of bias assessment, Questions 1 to 3

| **Study ID** | **1a.Were appropriate data sources used, e.g. cohort, CT or nested case-control study data?** | **1b.Were all inclusions and exclusions of participants appropriate?** | **1c.Were participants enrolled at a similar state of health, or were predictors considered to account for differences?** | **I. Participant selection** | **2a.Were predictors defined and assessed in a similar way for all participants in the study?** | **2b.Were predictor assessments made without knowledge of outcome data?** | **2c.Are all predictors available at the time the model is intended to be used?** | **2d.Were all relevant predictors analysed?** | **II. Predictors** | **3a.Was a pre-specified outcome definition used?** | **3b.Were predictors excluded from the outcome definition?** | **3c.Was the outcome defined and determined in a similar way for all participants?** | **3d.Was the outcome determined without knowledge of predictor information?** | **III. Outcome** |
| --- | --- | --- | --- | --- | --- | --- | --- | --- | --- | --- | --- | --- | --- | --- |
| Marshall 2011[29] | Yes | Yes | Yes | Low risk | Yes | Yes | Yes | Unclear | Unclear risk | Yes | Yes | Yes | Yes | Low risk |
| Fijten 1995[28] | No | Yes | Yes | High risk | Yes | Yes | Yes | Yes | Low risk | Yes | Yes | Yes | Yes | Low risk |
| Hodder 2005[52] | No | Unclear | Yes | High risk | Yes | Yes | Yes | Unclear | Unclear risk | Yes | No | Yes | No | High risk |
| Kop 2015[53] | Yes | Yes | Yes | Low risk | Yes | Yes | Yes | Unclear | Unclear risk | Yes | Yes | Yes | Yes | Low risk |
| Hippisley-Cox 2013[38] | Yes | Yes | Yes | Low risk | Yes | Yes | Yes | Yes | Low risk | Yes | Yes | Yes | Yes | Low risk |
| Hippisley-Cox 2013b[37] | Yes | Yes | Yes | Low risk | Yes | Yes | Yes | Yes | Low risk | Yes | Yes | Yes | Yes | Low risk |
| Collins 2012[23] | Yes | Yes | Yes | Low risk | Yes | Yes | Yes | Yes | Low risk | Yes | Yes | Yes | Yes | Low risk |
| Hippisley-Cox 2012c[22] | Yes | Yes | Yes | Low risk | Yes | Yes | Yes | Yes | Low risk | Yes | Yes | Yes | Yes | Low risk |
| Hamilton 2015[36] | Yes | Yes | Yes | Low risk | Yes | Yes | Yes | Yes | Low risk | Yes | Yes | Yes | Yes | Low risk |
| Stapley 2017[35] | Yes | Yes | Yes | Low risk | Yes | Yes | Yes | Yes | Low risk | Yes | Yes | Yes | Yes | Low risk |
| Hamilton 2005[33] | Yes | Yes | Yes | Low risk | Yes | Yes | Yes | Yes | Low risk | Yes | Yes | Yes | Yes | Low risk |
| Hamilton 2009[40] | Yes | Yes | Yes | Low risk | Yes | Yes | Yes | Yes | Low risk | Yes | Yes | Yes | Yes | Low risk |
| Elias 2017[27] | Yes | Yes | Yes | Low risk | Yes | Yes | Yes | Yes | Low risk | Yes | Yes | Yes | Yes | Low risk |
| Nørrelund 1996[31] | Yes | Yes | Yes | Low risk | Yes | Unclear | Yes | Unclear | Unclear risk | Yes | Yes | Yes | Unclear | Low risk |
| Muris 1995[30] | Yes | Unclear | Yes | Unclear risk | Yes | Yes | Yes | Yes | Low risk | Yes | Yes | Yes | Yes | Low risk |
| Holtedahl, 2018[39] | Yes | Yes | Unclear | Unclear risk | Yes | Yes | Yes | Yes | Low risk | Unclear | Yes | Unclear | Yes | Unclear risk |

Abbreviations: ID = identification

Table S3b. Development and validation studies - Risk of bias assessment, Questions 4 to 5

| **Study ID** | **4a.Were there a reasonable number of outcome events?** | **4b.Was the time interval between predictor assessment and outcome determination appropriate?** | **4c.Were all enrolled participants included in the analysis?** | **4d.Were participants with missing data handled appropriately?** | **IV. Sample size and participant flow** | **5a.Were non-binary predictors handled appropriately?** | **5b.Was selection of predictors based on univariable analysis avoided?** | **5c.Was model overfitting (optimism in model performance) accounted for, e.g. using bootstrapping or shrinkage techniques?** | **5d.Were any complexities in the data (e.g. competing risks, multiple events per individual) accounted for appropriately?** | **5e.Do predictors and their assigned weights in the final model correspond to the results from multivariable analysis?** | **5f.For the model or any simplified score, were relevant performance measures evaluated, e.g. calibration, discrimination, (re)classification and net benefit?** | **5g.Was the model recalibrated or was it likely (based on the evidence presented, e.g. calibration plot) that recalibration was not needed?** | **V. Analysis** |
| --- | --- | --- | --- | --- | --- | --- | --- | --- | --- | --- | --- | --- | --- |
| Marshall 2011[29] | Yes | Yes | Yes | Unclear | Unclear risk | Yes | Yes | Yes | Yes | Yes | Yes | Yes | Low risk |
| Fijten 1995[28] | Unclear | Yes | Yes | Yes | Unclear risk | Yes | No | No | Yes | Yes | Yes | No | High risk |
| Hodder 2005[52] | Yes | Yes | Yes | Yes | Low risk | Unclear | No | No | Yes | Yes | Yes | No | Unclear risk |
| Kop 2015[53] | Yes | Yes | Yes | Unclear | Unclear risk | Yes | Yes | Unclear | Unclear | Unclear | Unclear | Unclear | Unclear risk |
| Hippisley-Cox 2013[38] | Yes | Yes | Yes | Yes | Low risk | Yes | Yes | Yes | Yes | Yes | Yes | Yes | Low risk |
| Hippisley-Cox 2013b[37] | Yes | Yes | Yes | Yes | Low risk | Yes | Yes | Yes | Yes | Yes | Yes | Yes | Low risk |
| Collins 2012[23] | Yes | Yes | Yes | Yes | Low risk | Yes | Yes | Yes | Yes | Yes | Yes | Yes | Low risk |
| Hippisley-Cox 2012c[22] | Yes | Yes | Yes | Yes | Low risk | Yes | Yes | Yes | Yes | Yes | Yes | Yes | Low risk |
| Hamilton 2015[36] | Yes | Yes | Yes | Unclear | Unclear risk | Yes | No | Unclear | Yes | Yes | No | Unclear | High risk |
| Stapley 2017[35] | Yes | Yes | Yes | Unclear | Unclear risk | Yes | No | No | Yes | Yes | No | No | High risk |
| Hamilton 2005[33] | Yes | Yes | Yes | Unclear | Unclear risk | Yes | No | Unclear | Yes | Yes | No | Unclear | High risk |
| Hamilton 2009[40] | Yes | Yes | Yes | Unclear | Unclear risk | Yes | No | Unclear | Yes | Yes | No | Unclear | High risk |
| Elias 2017[27] | Yes | Unclear | No | Unclear | Unclear risk | Unclear | NA | NA | NA | NA | Yes | Unclear | Unclear risk |
| Nørrelund 1996[31] | Yes | Yes | Unclear | Unclear | Unclear risk | Unclear | Yes | No | No | No | Yes | No | High risk |
| Muris 1995[30] | No | Yes | Unclear | Unclear | Unclear risk | Yes | No | No | Unclear | Yes | No | No | High risk |
| Holtedahl, 2018[39] | Yes | Unclear | No | Unclear | High risk | Unclear | Yes | Unclear | Yes | Yes | Yes | No | Unclear risk |

Abbreviations: ID = identification

Table S4a. Impact Studies - Characteristics

| **Study ID** | **Prediction tool** | **Cancer type(s)** | **Country** | **Study design** | **Intended purpose** |
| --- | --- | --- | --- | --- | --- |
| Hamilton and colleagues, 2013[55] | RAT for lung cancer, CRC in two formats: mouse mat and desktop flip chart | Lung, CRC | UK | Cohort study | To compare referrals and investigations for colorectal and lung cancer before and after the implementation of RATs |
| Emery and colleagues, 2017[56] | Composite intervention including the RAT for colorectal, lung and prostate, as well as summaries of relevant guidelines for colorectal, lung, prostate and breast cancer | CRC, lung, prostate, BC | Australia | Factorial cluster RCT | To measure the effect of community-based symptom awareness and general practice-based educational interventions on the time to diagnosis in rural patients presenting with breast, prostate, colorectal or lung cancer in Western Australia |
| Price and colleagues 2019[16] | RAT, QCancer in any form (e.g. paper, software etc.) | Any type | UK | Cross-sectional survey | to explore the association between tool availability and 2-week-wait (2WW) referrals for suspected cancer |

Abbreviations: BC = breast cancer; CRC = colorectal cancer; GP(s) = general practitioner(s); ID = identification; NHS = National Health Service; RAT(s) = risk assessment tool(s); RCT = randomised controlled trial; SR1 = systematic review 1; UK = United Kingdom

Table S4b. Impact studies - Study Design

| **Study ID** | **Population** | **Recruitment (+ inclusion and exclusion criteria)** | **Sample size** | **Tool description** | **Predictors** |
| --- | --- | --- | --- | --- | --- |
| Hamilton and colleagues, 2013[55] | Number of investigations (x-rays or colonoscopies) and 2WW referrals from practices and local NHS trusts for the two 6-mth periods before and after the distribution of the tools | A selected GP cancer lead from 7 of the 28 English cancer networks recruited local GP practices to which the RATs were supplied. 614 GPs from 165 practices were recruited. 2,593 assessments were included | 614 GPs from 165 practices | RAT gives risk estimates for patients aged >40 yrs presenting to primary care with symptoms of possible cancer, for single symptoms, pairs of symptoms and repeat attendances with the same symptom. The values are colour-coded to aid interpretation. | For colorectal cancer: symptoms (constipation, diarrhoea, rectal bleeding, abdominal pain, abdominal tenderness, abnormal rectal exam), loss of weight, haemoglobin (10–13g/dl; <10g/dl); NR for other cancers |
| Emery and colleagues, 2017[56] | Two trial areas in Western Australia | Inclusion criteria: adults aged over 18 years; diagnosed with breast, lung, colorectal or prostate cancer between 1 Jan 2012 and the recruitment end date of 31 Mar 2014; and resident of trial areas at the time of cancer diagnosis. | 1,358 patients (497 in trial area A, 861 in trial area B) | Resource card containing the RAT tables for colorectal, lung and prostate cancer, as well as the National Breast and Ovarian Cancer Centre guidelines for investigating new breast symptoms | NR |
| Price and colleagues 2019[16] | GPs working in a general practice in UK | 975 UK randomly selected general practices | 476 GPs in 227 practices | Practice access to RAT and/or Qcancer | NR |

Abbreviations: GP(s) = general practitioner(s); mth(s) = month(s); NHS = National Health Service; NICE = National Institute for Health and Care Excellence; NR = not reported; PHE = Public Health England; RAT(s) = risk assessment tool(s); SR1 = systematic review 1; WW = week wait; yr(s) = year(s)

Table S4c. Impact studies - Results

| **Study ID** | **Outcomes (definitions)** | **Outcomes** | **Main results** |
| --- | --- | --- | --- |
| Hamilton and colleagues, 2013[55] | Investigation for cancer: 2-week referral to the appropriate speciality, or a chest X-ray in possible lung cancer | Number of investigations and two-week referrals | Lung cancer: 31% increase in 2-week referrals (332 before, 436 after); 4% increase in related investigations (chest X-ray) (7431 before, 7723 after) Colorectal cancer: 26% increase in 2-week referrals (1,173 before, 1,477 after); 15% increase in colonoscopies (1,762 before, 2,032 after) |
| Emery and colleagues, 2017[56] | TDI, as the time from first symptom to cancer diagnosis | Time to diagnosis interval (TDI) | No significant differences in the median or ln mean TDI at either intervention level: GP intervention vs control: median TDI 97 vs 96.5 days; ln mean difference 0.004 95% CI 0.18–0.19 P¼0.99).  community intervention vs control: median TDI 107.5 vs 92 days; ln mean difference 0.08 95% CI 0.06–0.23 P=0.27; no significant differences in the TDI when analysed by factorial design, tumour group or sub-intervals of the TDI. |
| Price and colleagues 2019[16] | Referral activity was measured using the practices’ age- and sex-adjusted numbers of 2WW referrals for suspected cancer per 100 000 of the population | Referral rate | no difference in mean 2WW referral rate between practices with or without access to either type of tool, after adjusting for Index of Multiple Deprivation (mean difference 3.1 referrals per 100 000 [95% CI = –5.5 to 11.7] per 100 000, P = 0.478) |

Abbreviations: ANOVA = analysis of variance; CI = confidence interval; GP(s) = general practitioner(s); ID = identification; mth(s) = month(s); NICE = National Institute for health and Care Excellence; SR1 = systematic review 1; TDI = total diagnostic interval; WW = week wait; yr(s) = year(s)

Table S5. Impact studies - Critical Appraisal

| **Study ID** | **Random sequence generation** | **Allocation concealment** | **Baseline outcome measurements similar** | **Baseline characteristics similar** | **Incomplete outcome data** | **Knowledge of the allocated interventions adequately prevented during the study** | **Protection against contamination** | **Selective outcome reporting** | **Other risks of bias** |
| --- | --- | --- | --- | --- | --- | --- | --- | --- | --- |
| Hamilton and colleagues, 2013[55] | N/A (cohort study) | N/A (cohort study) | N/A (cohort study) | N/A (cohort study) | Unclear risk (details not reported) | High risk (the GPs were not blind, irrespective of whether they were counted or not) | N/A (cohort study) | Unclear risk | High risk (other important influencing factors could have occurred during the before and after. Unclear how many GPs involved) |
| Emery and colleagues, 2017[56] | Low risk (allocation of interventions was random) | High risk (allocation not concealed) | Low risk | Low risk | Low risk | Low risk (research staff who collected outcome data and the trial statistician were blinded to group allocation) | Low risk (measures were taken to avoid contamination: re-clustering of practices attended by same practitioners, use of media avoided in the control areas) | Low risk | Low risk |
| Price and colleagues 2019[16] | N/A (observational study) | N/A (observational study) | N/A (observational study) | Unclear risk (analyses adjusted for deprivation index) | Unclear risk (details not reported) | Low risk (data on referrals was collected independently from the survey) | N/A (observational study) | Low risk | High risk (small sample, access to tools self-reported) |

Abbreviations: GP(s) = general practitioner(s); ID = identification; N/A = not applicable; RCT = randomised controlled trial; SR1 = systematic review 1
